# Embryonic and Larval Development and Early Behavior in Grass Carp, *Ctenopharyngodon idella*: Implications for Recruitment in Rivers

**DOI:** 10.1371/journal.pone.0119023

**Published:** 2015-03-30

**Authors:** Amy E. George, Duane C. Chapman

**Affiliations:** U.S. Geological Survey—Columbia Environmental Research Center, 4200 New Haven Road, Columbia, MO 65201, United States of America; Central Michigan University, UNITED STATES

## Abstract

With recent findings of grass carp *Ctenopharyngodon idella* in tributaries of the Great Lakes, information on developmental rate and larval behavior is critical to efforts to assess the potential for establishment within the tributaries of that region. In laboratory experiments, grass carp were spawned and eggs and larvae reared at two temperature treatments, one “cold” and one “warm”, and tracked for developmental rate, egg size, and behavior. Developmental rate was quantified using Yi’s (1988) developmental stages and the cumulative thermal units method. Grass carp had a thermal minimum of 13.5°C for embryonic stages and 13.3°C for larval stages. Egg size was related to temperature and maternal size, with the largest eggs coming from the largest females, and eggs were generally larger in warmer treatments. Young grass carp larvae exhibited upward and downward swimming interspersed with long periods of lying on the bottom. Swimming capacity increased with ontogeny, and larvae were capable of horizontal swimming and position holding with gas bladder emergence. Developmental rates, behavior, and egg attributes can be used in combination with physical parameters of a river to assess the risk that grass carp are capable of reproduction and recruitment in rivers.

## Introduction

Asian carps (silver carp *Hypophthalmichthys molitrix*, bighead carp *H*. *nobilis*, grass carp *Ctenopharyngodon idella*, and black carp *Mylopharyngodon piceus*) are invasive species in North American waterways, and have spread through the Mississippi River system to the Laurentian Great Lakes area. Large-scale projects including the use of electric barriers, environmental DNA monitoring, and seismic technology are being investigated and employed to keep these fish out of the Great Lakes and to prevent damage to a fishery valued at over $7 billion dollars [1, 2, 3].

The eggs of Asian carps develop to hatching while drifting in river currents. The eggs are slightly heavier than water and are kept in suspension through the turbulence of the flowing water. They will settle to the bottom if turbulence drops below a critical value. It has long been thought that settlement is lethal to Asian carp eggs [4, 5], though this assertion is based on circumstantial evidence with no known mechanism. Field observations have cited the length of river necessary for successful reproduction and recruitment of Asian carp as anywhere from 28 to 100km; [5, 6, 7]. Garcia et al. [8] produced a model of dispersal distances for bighead carp and silver carp (the bigheaded carps) eggs, based on physical data from specific rivers and developmental rates and egg attributes of the bigheaded carps, and found that a river length of 14.5 km would be sufficient for recruitment under certain conditions. Using the model, Murphy and Jackson [9] found that bigheaded carp eggs would have sufficient river length for hatching in the Sandusky River, a tributary of Lake Erie. Following this prediction, grass carp were found to have successfully recruited in that river [10], the first evidence of any Asian carp recruitment in the Great Lakes Basin. An exact river length sufficient for egg transport is dependent on factors such as water velocity, turbulence, temperature, and species-specific biological data, and will thus vary for different rivers, times, and species. Computing drift distances and length of river necessary for successful spawning is critical for efforts to determine the potential for Asian carp recruitment in rivers, and thus assessing the invasion risk to the Great Lakes or to reservoir systems. Determining the locations where grass carp recruitment can occur may be useful in developing control efforts.

Developmental rates and early behavioral information for grass carp are sparsely documented relative to conditions commonly found in North American waterways. Yi et al. [11] provide a complete description of grass carp developmental stages, although this work lacks temperature controls [12]. Korwin-Kossakowski [13] provides temperature controlled data, although mostly at higher temperatures and with fewer developmental stages. Developmental rates in poikilotherms are highly correlated with temperature, and even small changes in temperature can drastically affect those rates. At higher temperatures, embryos develop quickly, thus requiring a much shorter length of river than embryos produced at lower temperatures. The first objective of this work was to determine the rate for grass carp embryonic and larval development using the cumulative thermal units (CTU) methodology, to provide data necessary to develop egg drift models for grass carp that are similar to those that have been created for bigheaded carps. Secondly, we sought to describe egg size over the embryonic period. Our final objective was to describe the early larval behavior of grass carp. Observations from these three areas are examined within the context of egg and larvae drift and dispersal from the spawning site.

## Materials and Methods

### Ethics statement

The study plan was approved by the Columbia Environmental Research Center Institutional Animal Care and Use Committee and conforms to relevant national and international guidelines. Larval grass carp were anesthetized to death using tricaine methanesulfonate (MS-222). Adult grass carp used as brood stock were obtained using trammel nets from the Missouri River under Missouri Department of Conservation permits and overwintered in Columbia Environmental Research Center (CERC) ponds. One large female used for the third trial was captured by trammel net in J. Fairchild’s private farm pond.

Water from CERC wells was used for all experiments. In each round of experiments, two temperature treatments were used to culture eggs and larvae. Each temperature treatment consisted of 6 aquaria, which were equipped with a modified MacDonald hatching jar (45 cm tall and 13 cm internal diameter) and a small submersible pump to recirculate water from the aquarium into the hatching jar, and to provide an upwelling current to keep the eggs suspended in the water column within the jar. Jars were equipped with surface screens to prevent any eggs escaping into the aquarium before hatching. All aquaria were provided with a slow flow-through of water.

Temperature was maintained in water baths by the use of heaters and chillers. Temperature was monitored continuously with HOBO temperature loggers (Onset Computer Company, model Pro-v2) recording at 15 minute intervals. The temperature range of 19–23°C was selected to reflect conditions that are common in North American mid-continent rivers, and are not intended to represent thermal minima, maxima, or optima. For the first trial, the mean warm temperature was 22.75°C, with a standard deviation of 0.53°C and the mean cold temperature was 19.03 ± 0.19°C. In the second trial, the mean warm temperature was 22.38 ± 0.16°C and the mean cold temperature was 19.0 ± 0.06°C. In the third trial, the mean warm temperature was 22.57 ± 0.28°C and the mean cold temperature was 19.21 ± 0.10°C.

### Aquaculture methods

Fish were collected from ponds and evaluated for spawning readiness, using 50 mg/L MS-222 as anesthesia. Males were detected by the presence of milt after the application of gentle abdominal pressure, and females were evaluated by catheter insertion into the urogenital pore to collect oocyte samples. Oocytes were evaluated for readiness on consistency of size, color, shape and the migration of the germinal vesicle (GV). To determine GV migration, oocytes were placed into Pankurst clearing solution (60% ethanol, 30% formalin, 10% acetic acid) to ascertain whether the GV had migrated towards the animal pole. Fish determined to be ready for spawning were held in 2100-liter flow-through poly tanks supplied with CERC pond water. Temperatures in these tanks were normally maintained between 21.5–23.8°C, although there was a temperature spike of up to 29.3°C with the third set of spawning fish.

Spawning was induced in both males and females according to Jhingran and Pullin [14]. Male grass carp received a 3 mg/kg intramuscular carp pituitary injection 24 hours prior to the expected ovulation period. Milt was collected into plastic tubes and stored for 2–4 hours in beakers on ice prior to fertilization. Quality of the milt was evaluated by checking percent of active sperm and duration of motility. Females received 1–2 intramuscular injections of human chorionic gonadotropin (HCG) based on fish weight, temperature, and spawning readiness, and resolving doses (given as intramuscular injections) of 8.8 mg/kg carp pituitary gland were given 24 hours after the other injections.

#### Trial 1

Two female grass carp (4.0kg and 7.0kg) and five male grass carp (2.7, 4.4, 4.4, 4.5, and 4.8 kg) were selected for hormone induction. The females initially received an intramuscular injection of 200 IU/KG HCG, and received a second intramuscular injection of 1200 IU/KG HCG 12 hours later.

#### Trial 2

Two female grass carp (3.2 kg and 4.4 kg) and four male grass carp (3.3, 4.7, 4.8, and 6.4 kg) were selected for hormone induction. The females received an initial intramuscular injection of 1870 IU/KG HCG.

#### Trial 3

Two female grass carp (6.1 kg and 18 kg) and five male grass carp (4.9, 5.3, 5.5, 5.9, and 6.7 kg) were selected for hormone induction. Females initially received an intramuscular injection of 1500 IU/KG HCG.

Ova were stripped into a bowl 30 minutes after the application of gentle abdominal pressure caused the initial release of eggs, and were then fertilized with pooled milt by the dry method [15] for one minute (with a ratio of approximately 1 mL milt: 1 g eggs), then rinsed and placed into a water bath for a 30-minute water-hardening period. Two water baths were used, matching the temperature in the hatching jars where the eggs were stocked. With multiple females, eggs were generally combined during fertilization, although in the third trial, the eggs from each female were kept separate throughout development because of a large size difference between the two females. After approximately one hour of water hardening, 100mL of eggs (approximately 5,000 eggs) were moved from the water baths into each of the hatching jars.

Three to six eggs were initially removed from tanks at 15 minute intervals. After water hardening (expected to last 4 hours), the interval for removal increased to 30 minute; after hatching there was a 4-hour interval for larval removal. Imaging and all size measurements were done on live specimens, using a Nikon SMZ1500 microscope and NIS Elements software for image analysis. Specimens were preserved in 10% formalin, and stage assessment and further imaging were done later. Stages were assessed according to Yi et al. [11] and are illustrated in the supporting information of this article (S1 File). In all trials, no efforts were made to quantify the degree of mortality.

### CTU methodology

Cumulative Thermal Units (CTU), also known as degree-days or temperature units, is a method designed to calculate the relation between temperature and developmental time in poikilotherms. As in George and Chapman [16], the following equation was used for calculating CTU:

$$CTU=t\left(T_{c}-T_{min}\right)$$

where t = time in hours, T_c_ = treatment temperature in degrees Celsius, and T_min_ = thermal minimum in degrees Celsius.

Using all experimental data for each species, CTU was computed iteratively for each developmental stage, by using a value for T_min_ from 0.0°C to 15.0°C in 0.1°C increments, in order to determine the value of T_min_ where the CTU value had the lowest variance for all developmental stages. Variance was tested using a one-way ANOVA with stage as the parameter. The main purpose was to obtain the pooled variance and establish the coefficient of determination (R^2^). To obtain the best fit, T_min_ values are reported as separate values for embryonic stages and larval stages.

### Larval behavior

Twenty newly hatched larvae were placed in each of two vertical tubes (2m high, 9.4cm internal diameter), similar to those used in George and Chapman [16] but composed of clear butyrate. Temperature in the behavioral tubes was controlled by room temperature and monitored with a HOBO temperature logger. All behavioral experiments were stocked with larvae from the warmer treatment. In an otherwise light-tight room, lamps placed at the top of the swimming tubes provided a photoperiod of 14 hours light: 10 hours dark. This period was selected to imitate the peak spawning season of Asian carps. Water column position, swimming patterns, and ascent and descent rates were observed at 12 hour intervals, including one dark period and one light period measurement. Small flashlights and indirect illumination were used for observation of fish during dark period. During the period of development when larvae exhibited only vertical swimming, water column position represents the starting point of a vertical ascent. During the period after emergence of the gas bladder when larvae were able to maintain vertical position through buoyancy compensation or horizontal swimming, it represents a depth where the larvae was located for at least 5 seconds. Ascent and descent rates were measure for each fish, and mean ascent and descent rates of all fish are reported for each interval during the vertical swimming period. Developmental stage of larvae was determined by visual inspection, and observations continued until after gas bladder inflation, a period of at least 6 days.

Approximately 20 larvae were also placed in a 2-gallon aquarium to observe other behaviors, including responses to light and small-scale turbulence (provided by an air bubbler). Half of the tank was shaded with black plastic, and the other half was illuminated by a small lamp with a 60-watt light bulb. Descriptive observations were made at the same intervals as the swimming tubes.

### Egg size

Eggs were not exactly spherical, therefore two perpendicular diameters were taken on each measured egg and the mean value used. Water hardening time was determined using a piecewise two-segment regression for each temperature treatment. A student’s two-tailed t-test was performed to compare the diameter of water hardened eggs between the two temperature treatments. Significance was established at α = 0.05.

## Results

### Water Quality

Water quality readings were well within normal parameters for grass carp rearing. Ammonia readings ranged from 0.017–0.63 ppm as NH^3-^N. Water hardness was 260–304 mg/L as CaCO_3_, and alkalinity was 224–260 mg/L as CaCO_3_. The pH was between 7.45 and 8.38 in all treatments, with a mean of 7.96 ± 0.149. Conductivity was 635–693 μS/cm. Dissolved oxygen was between 5.74 and 9.04 mg/L, and total dissolved solids were between 409.5 and 448.5mg/L.

### Development rates and stages

A T_min_ of 13.5°C for eggs (R^2^ = 0.9954, root MSE = 5.3477), and 13.3°C for larvae (R^2^ = 0.9899, root MSE = 36.0138) provided the lowest variance in CTUs (Table 1).

**Table 1:** Cumulative thermal units (CTU) for grass carp development at various stages. T_min_ is given separately for embryonic and larval stages.

Stage		Average	Standard Deviation
		T_min_ = 13.5	
1	1-cell	0.422	0.163
2	2-cell	7.774	0.670
3	4-cell	9.915	0.988
4	8-cell	13.034	1.177
5	16-cell	17.737	2.764
6	32-cell	22.113	2.604
7	64-cell	27.576	2.167
8	128-cell	32.526	2.948
9	Morula	40.119	4.679
10	Early blastula	49.851	6.261
11	Mid-blastula	57.001	5.511
12	Late blastula	64.641	4.008
13	Early gastrula	73.497	4.170
14	Mid gastrula	82.131	5.159
15	Late gastrula	93.379	6.260
16	Neurula	106.730	3.315
17	Blastopore closure	115.341	3.212
18	Somite appearance	123.923	4.539
19	Optic primordium	131.428	2.638
20	Optic vesicle	140.683	3.197
21	Olfactory placode	148.861	5.415
22	Tail bud	155.705	4.719
23	Otic capsule	164.837	3.697
24	Tail vesicle	174.921	6.665
25	Caudal fin	184.279	3.754
26	Lens formation	193.909	4.446
27	Muscular effect	207.621	8.476
28	Heart rudiment	219.315	8.140
29	Otolith appearance	229.581	12.617
30	Heartbeat	241.097	11.690
		T_min_ = 13.3	
31	Hatching	258.452	12.391
32	Rudimentary pectoral fin	389.714	27.343
33	Gill arches	479.099	34.886
34	Xanthic eye	567.081	55.709
35	Gill filaments	684.696	36.486
36	Melanoid eye	866.577	31.150
37	Gas bladder emergence	987.968	49.853
38	One chamber gas bladder	1100.820	25.939

### Egg Size

In trial 1, the water-hardened eggs from the warm treatment had a mean diameter of 4.42 ± 0.25 mm, while the eggs from the cold treatment were 4.02 ± 0.28 mm in diameter (t = -14.47, df = 408, P < 0.001). In trial 2, water hardened eggs from the warm treatment had a mean diameter of 4.13 ± 0.41mm and eggs from the cold treatment were 3.77 ± 0.34mm (t = -9.196, df = 380, P < 0.001). In trial 3, the mean water hardened diameter of eggs from the largest female was 4.65 ± 0.22mm in the warm treatment and 4.64 ± 0.29mm in the cold treatment (t = -0.357, df = 364, P = 0.72), while the smaller female had a mean water hardened diameter of 4.14 ± 0.22mm in the warm treatment and 4.10 ± 0.23mm in the cold treatment (t = -2.168, df = 356, P = 0.031).

**Fig 1:**
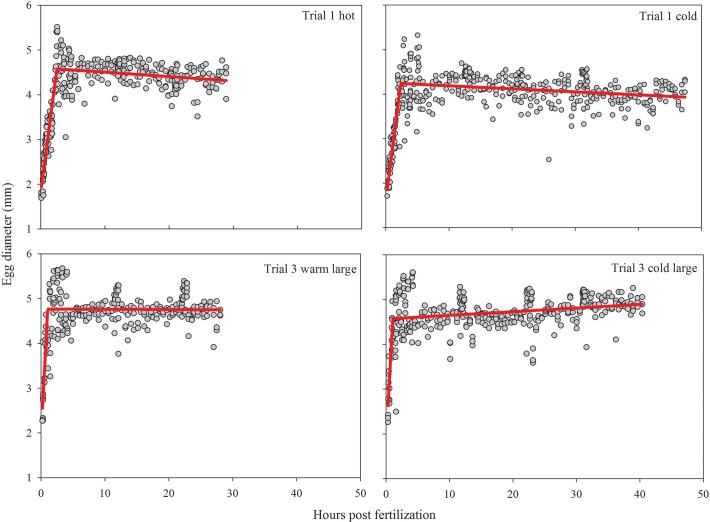
Grass carp egg diameter (mm) for both temperature treatments of trials 1 and 3 large. Trial 2 followed the same general trends as trial 1 and trial 3 small was similar to trial 3 large. The breakpoint of the red line indicates where water hardening occurs and eggs stop swelling.

Water hardening took approximately 2.5 hours for eggs in trial 1 (Warm, 2.35 hours: F_3,429_ = 355.12; P<0.0001; Cold, 2.5 hours: F_3,296_ = 526.19; P<0.0001), and the warm treatment of trial 2 (2.48 hours: F_3,273_ = 183.58; P<0.0001). Cold treatment eggs in trial 2 took 2.13 hours to water harden (F_3,392_ = 166.02; P<0.0001). Eggs from both fish and temperature treatments in trial 3 took approximately one hour to water harden (Large cold, 1.05 hours: F_3,343_ = 102.76; P<0.0001; Large Hot, 1.05 hours: F_3,255_ = 90.68; P<0.0001; Small cold, 0.96 hours: F_3,299_ = 142.08; P<0.0001) (Fig. 1).

### Behavior

For trial 1, temperature in the behavioral tubes varied between 22.0 and 23.2°C, with a mean temperature of 22.7 ± 0.20°C. In trial 2, temperature varied between 22.0 and 24.0°C, with a mean temperature of 23.6 ± 0.49°C. In trial 3, temperature in the behavioral tubes ranged between 20.0 and 25.1°C, with a mean of 22.6 ± 0.7°C. Temperatures did not differ between the top and bottom of the tube, and there was no significant difference in temperature between the tubes.

**Fig 2:**
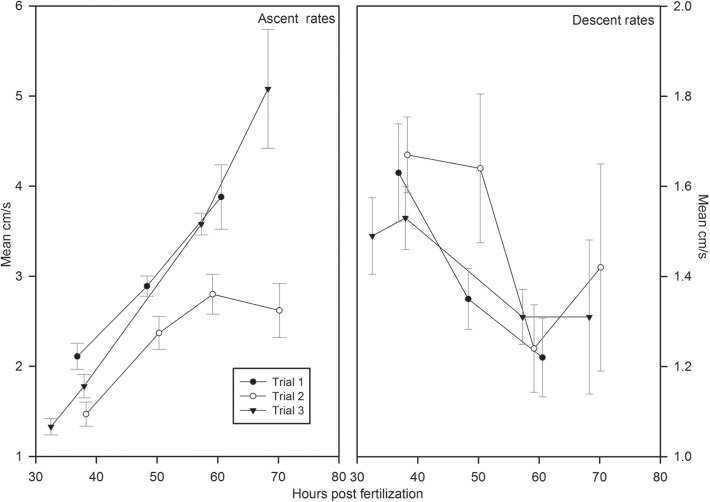
Mean ascent and descent rates (cm/s) for each of the three trials during the vertical swimming period. Error bars represent standard error.

**Fig 3:**
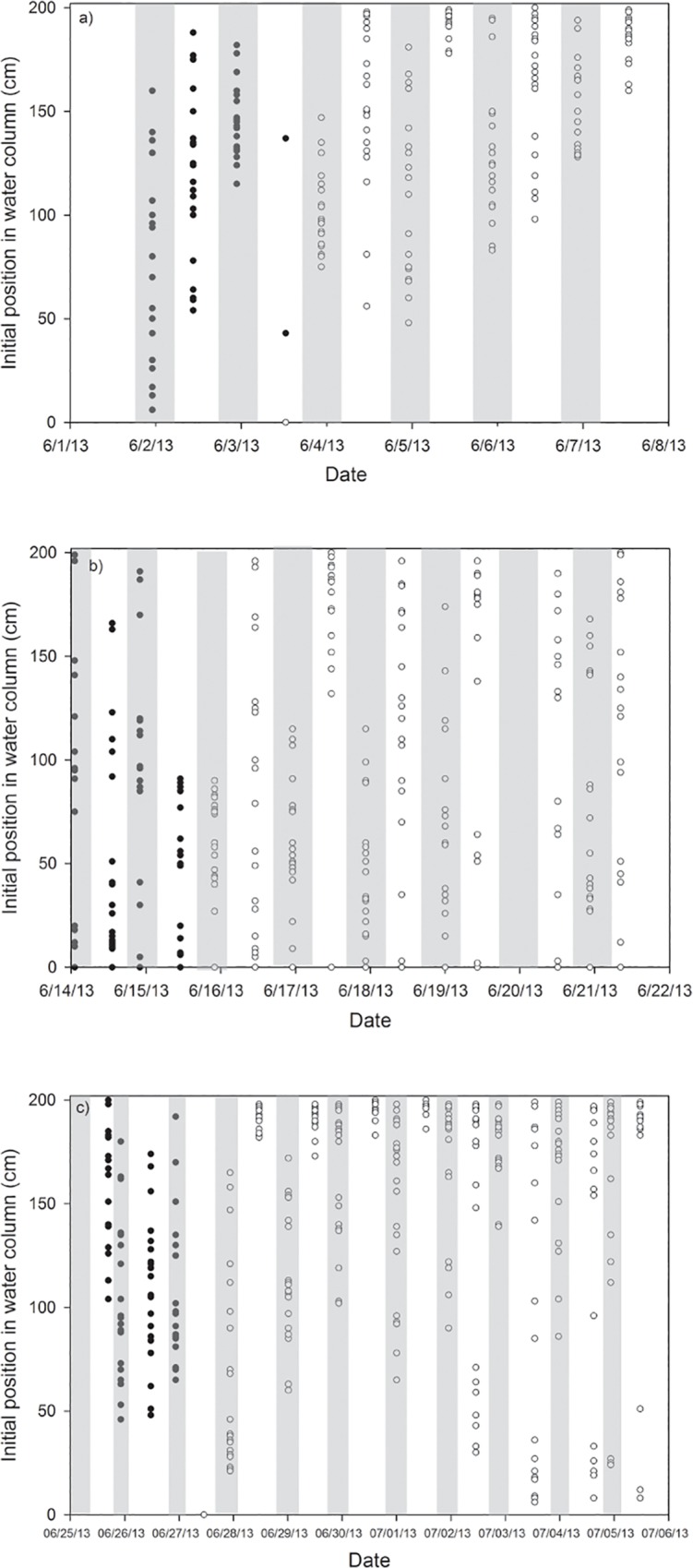
Initial water column location (cm from bottom) for larvae over time. Shaded areas represent night/dark hours. Dark circles represent vertically swimming larvae and light circles represent horizontally swimming larvae.

Grass carp larvae began vertical swimming soon after hatching. The larvae swam upwards for a period of several seconds, and would either fall or swim downwards. Upward swimming generally lasted slightly longer than downward movement. Mean total ascent was between 5 and 22 cm, and mean total descent was between 4 and 29 cm, depending on the trial and developmental stage. Ascent distances showed no clear patterns with ontogeny or diel cycle. Mean descent distances decreased with ontogeny. During the vertical swimming period, ascent rates increased with ontogeny while descent rates decreased (Fig. 2). During daytime hours, larvae were usually higher in the water column, particularly after the onset of horizontal swimming, and tended to be lower and more spread out during night (Fig. 3A, 3B, and 3C).

Larval fish also displayed long periods of lying on the bottom of the aquarium during larval stages 31–37, though most notably during the transition to gas bladder emergence. Horizontal swimming began in conjunction with the gas bladder emergence stage; at this point, the larvae could maintain depth and position. Larvae were also capable of vertical and horizontal swimming in limited current (provided by air bubbler). When a portion of the aquarium was shaded, larvae with horizontal swimming capabilities (i.e. stage 37 and above) exhibited mass movement (greater than 80% of larvae) into lighted areas.

## Discussion

### Developmental rates

Despite critiques, variants of the CTU model have been in use for over a century and have been found to predict incubation times with sufficient accuracy for most fishery management applications [17, 18]. Optimally, CTU is applied in conditions with relatively constant temperatures that are not near the thermal minima or maxima for a species or population. Large temperature fluctuations can affect developmental rate and other processes in unpredictable ways [18, 19, 20, 21];, and were not examined in this study. Early life history stages often show less tolerance to temperature extremes than adult or juvenile fish [21], and thus reported adult grass carp thermal limits of 33–42°C maximum and less than 0°C minimum [22, 23, 24, 25]) were not approached in this study. Temperatures were within normal ranges reported for grass carp spawning [26]. Asian carp spawning seems to be initiated by a combination of river discharge and temperature conditions [4], and this tends to ensure that spawning will take place in the optimal temperature range. While strong and fast temperature fluctuations can occur in large rivers, these are not the norm over the comparatively short embryonic period of grass carp. When such temperature fluctuations do occur, mortality rates tend to be very high for many species of fish [27].

T_min_ varied among developmental stages, although given the relatively small degree of change and rapid progression between stages, it is logical to use one T_min_ for all embryonic stages and another for all larval stages. The thermal minimum is also influenced by maternal history, acclimation temperature, population or stock differences, and environmental factors [28, 29]

Korwin-Kossakowski [13] reported similar times for several of the grass carp developmental stages (i.e. hatching and blastopore closure) at similar temperatures (20 and 24°C). However, his work was conducted at mostly higher temperatures and lists fewer developmental stages. Abdusamadov [30] reported slightly longer hatching time at similar temperatures in the Terek River. With stock and experimental temperatures selected from mid-continent North America, the use of the CTUs and T_min_ given in this paper is most appropriate within that geographical context. CTU requirements are consistent with needs for silver carp and bighead carp [16].

### Egg Size

It has long been thought that Asian carp eggs must stay suspended in the water column, or they will perish, although the mechanism for death has not been clear [4]. After the release of ova (and presumably fertilization), eggs rapidly absorb water and become water hardened. The density of eggs thus becomes closer to that of water and the eggs have a greater propensity to remain suspended in the water column, which is aided by turbulence. The degree of turbulence necessary to transport pre- and post-water hardened eggs can be computed. Water-hardened eggs, which are larger and less dense, can likely be transported in lower velocity and less turbulent rivers than pre-water hardened eggs. Water hardening does not seem to be affected by fertilization state, developmental stage, or temperature.

Egg diameter reported here for the post-water hardened period is smaller than reported in other literature [11, 31]]. Those studies used wild captured eggs, or laboratory spawned fish with no size details given. Egg size is dependent on a number of factors including water quality, temperature, maternal size, salinity, and ionic composition of the water [32, 33, 34]. As previously reported in silver and bighead carp [16], eggs in warmer water were generally larger than eggs in cooler water. In this study, maternal size and temperature seem to have had the largest effect on egg size. The largest grass carp (18.1kg) in this study produced distinctly larger eggs, with size unaffected by temperature. Grass carp eggs are generally intermediate in size between silver carp eggs and bighead carp eggs [11, 16].

Water hardening was faster in grass carp than in bighead carp or silver carp [16], and eggs in the third trial were much faster to water harden than eggs in any other trial. This indicates that there is less potential for settlement during the early period when eggs are the most dense and have the fastest sinking rate. Asian carps select areas of high turbulence for spawning [7], probably to promote transport of the eggs until they are water hardened. The faster water hardening rate of grass carp may give grass carp a greater potential for successful recruitment if high turbulence spawning areas are limited in length.

### Behavior

Grass carp larvae at the gas bladder emergence stage seemed to exhibit a very strong positive phototactic response, indicating that light traps could be an effective means of sampling grass carp larvae, and possibly juveniles. Stages before the development of the melanoid eye did not exhibit any phototactic behavior, although it is not clear whether that is a result of lack of eye development or swimming ability. It is common in many species of fish for the degree and type of phototaxis to change with ontogeny [35, 36], although we did not quantify this behavior past pro-larval stages. With developmental stage specific positive phototaxis, success with light traps will be largely dependent on timing and the selection of appropriate habitat for sampling. Positive phototaxis first occurred in grass carp at the gas bladder emergence phase (approximately 3–4 days post fertilization in the warmer trials), and more observation at later developmental stages is necessary to determine the time period that light traps would be effective sampling gear. Past collections of grass carp larvae (notably Conner et al. [37] and Brown and Coon [38]) have not used light traps as sampling gear.

The ascent and descent rates of vertically swimming larvae reported here could be incorporated in particle transport models that would help determine drift distance and location in the water column. Larvae were capable of vertical swimming in aquaria with light current from hatching, although this behavior was unquantified. In a fluvial system, larvae would likely be capable of interacting with currents after hatching, and this interaction would influence drift distance. Larval behavior, swimming speed, and activity levels are often influenced by temperature [39, 40, 41], although we did not specifically test for these effects in this experiment.

The phototactic response, and the movement of grass carp larvae nearer to the surface in light periods, may also be important for design and interpretation of collections based on ichthyoplankton tows; the distribution of fish will be dependent on the time of sampling. Also, in fluvial systems, flow rate is highest near the surface [42]. A strong phototactic response will keep larvae higher in the water column during daylight hours and thus subject to the increased velocity of the currents near the surface [43], and water column location selected by larvae can potentially influence developmental rate and drift distance.

### Drift distance and settling

Turbulence (shear velocity), not linear velocity, is the key factor that determines whether an egg of a given density and settling rate will be transported in a river or settle to the bottom [9]. Turbulence is dependent on linear velocity, depth, substrate rugosity and bedform. However, linear velocity is much easier to measure and several authors [5, 30, 44, 45] [5, 30] have proposed minimum linear velocities required for transport of the eggs of Asian carps. With the exception of Leslie et al. [5], these minimum velocities were based on known locations of Asian carp establishment, not on experimental data. Leslie et al. [5] found that a flow of 0.23 m/s was sufficient for the transport of unfertilized water hardened grass carp eggs, but that study was performed in a small stream and turbulence was probably greater than would be experienced for a similar velocity in the larger rivers where Asian carps commonly spawn (see [9]). Kocovsky et al. [46] determined that many of the tributaries to Lake Erie had sufficient length and temperature regime for successful spawning of Asian carp, using a flow of 0.70 m/s as a minimum linear velocity for egg transport. This may not be the optimal flow rate for large-scale recruitment, but it does allow for transport of eggs in rivers previously thought at low risk.

Developmental time is largely dependent on temperature. A temperature difference of less than three degrees Celsius resulted in a 16–17 hour difference in hatching time and a nearly three day difference in time to gas bladder inflation. Egg size is often influenced by temperature, with warmer temperatures generally resulting in larger eggs that take less turbulence and lower flow velocity to remain in suspension. Behavior can be affected by temperature. Physical properties of water, such as density and viscosity are affected by temperature, which further affects swimming behavior and buoyancy of eggs and larvae [21]. Other biological processes, such as growth and metabolic functions, are also affected by temperature. Although all of these processes and factors are more likely to have a greater effect when considering a wider range of temperatures than in this study, the effect of temperature on time to various developmental stages, and thus to drift distances in rivers, is extremely important.

Although expert opinion is variable on the matter [47], structured risk assessments of grass carp in the Laurentian Great Lakes Basin have uniformly resulted in an assessment of high risk of establishment and undesirable effects [46, 47, 48, 49]. Furthermore, grass carp have the ability to colonize reservoir systems which have tributaries that provide adequate conditions for recruitment [50], and to colonize many rivers across the United States. Models can be developed from the data developed in this study that can be used to determine which rivers and tributaries are at risk for establishment by grass carp. The FluEgg model [8] was developed using similar data for bigheaded carps. Murphy and Jackson [9] used that model to predict that in the Sandusky River, the available length of 25 kilometers was sufficient for successful recruitment of bigheaded carps. Data presented in this paper will enable similar models to be created for grass carp. Such models have other potential uses in efforts to monitor and control grass carp. In conjunction with hydraulic data and information on spawning time and location, these models can allow better selection of sampling sites for drifting eggs and larvae. Where grass carp eggs or early larvae are collected in ichthyoplankton surveys, these data can also be used in drift models to locate spawning areas, as in Deters et al. [51], or to predict where larvae will exit the drift to their nursery areas in wetlands. With this knowledge, efforts to disrupt spawning events, to deter larvae from adequate nursery areas, or to modify nursery areas to prevent recruitment could be initiated. In addition, it may be possible to use these models to design settling areas downstream of spawning sites that would disrupt drift and result in egg mortality.

## Supporting Information

S1 File Illustrated embryonic and larval development in grass carp. (DOCX)

## References

[1] Taylor RM, Pegg MA, Chick JH (2005) Response of bighead carp to a behavioural fish guidance system. Fish Mgmt Ecol 12: 283–286.

[2] Southwick Associates Inc. (2008) Today’s angler Fernandina Beach, Florida: Southwick Associates, Inc. 206 p.

[3] Jerde CL, Mahon AR, Chadderton WL, Lodge DM (2011) “Sight-unseen” detection of rare aquatic species using environmental DNA. Conserv Letters 4: 150–157.

[4] Kolar CS, Chapman DC, Courtenay WR, Housel CM, Williams JD, et al (2007) Bigheaded carps: a biological synopsis and risk assessment Bethesda, Maryland: American Fisheries Society. 204 p.

[5] Leslie AJ, Van Dyke JM, Nall LE (1982) Current velocity for transport of grass carp eggs. Trans Am Fish Soc 111: 99–101.

[6] Krykhtin ML, Gorbach EI (1981) Reproductive ecology of the grass carp, Ctenopharyngodon idella, and the silver carp, Hypophthalmichthys molitrix, in the Amur Basin. J Ichthy 21(2):109–123.

[7] Nico LG, Williams JD, Jelks HL (2005) Potential geographic range Black Carp: Biological synopsis and a risk assessment of an introduced fish. Bethesda, Maryland: American Fisheries Society pp. 197–242.

[8] Garcia T, Jackson PR, Murphy EA, Valocchia AJ, Garcia MH (2013) Development of a Fluvial Egg Drift Simulator to evaluate the transport and dispersion of Asian carp eggs in rivers. Ecol Mod 263:211–222.

[9] Murphy EA, Jackson PR (2013) Hydraulic and water-quality data collection for the investigation of Great Lakes tributaries for Asian carp spawning and egg-transport suitability. U.S. Geological Survey. Scientific Investigations Report 2013–5106. 30 p.

[10] Chapman DC, Davis JJ, Jenkins JA, Kocovsky PM, Miner JG, et al (2013) First evidence of grass carp recruitment in the Great Lakes Basin. J Great Lakes Res 39:547–554.

[11] Yi B, Liang Z, Yu Z, Lin R, He M (1988) A comparative study on the early development of grass carp, black carp, silver carp, and big head of the Yangtze River In: Yi B, Yu Z, Liang Z, editors. Gezhouba water control project and four famous fishes in the Yangtze River, China. Wuhan, China: Hubei Science and Technology Press pp. 69–135.

[12] Chapman DC, Wang N (2006) Early development of four cyprinids native to the Yangtze River, China: U.S. Geological Survey Data Series 239, 51p.

[13] Korwin-Kossakowski M (2008) The influence of temperature during the embryonic period on larval growth and development in carp, *Cyprinus carpio* L., and grass carp, *Ctenopharyngodon idella* (Val.): theoretical and practical aspects. Arch Polish Fish 16(3):231–314.

[14] Jhingran VG, Pullin RSV (1985) A hatchery manual for the common, Chinese and Indian major carps Manila, Philippines: Asian Development Bank, Philippines and International Center for Living Aquatic Resources Management. 191p.

[15] Piper RG, McElwain IB, Orme LE, McCraren JP, Fowler LG, et al (1982) Fish hatchery management Washington, D.C.: U.S. Fish and Wildlife Service.

[16] George AE, Chapman DC (2013) Aspects of embryonic and larval development in bighead carp *Hypophthalmichthys nobilis* and silver carp *Hypophthalmichthys molitrix* . PLoS One 8: e73829 doi: 10.1371/journal.pone.0073829 23967350 10.1371/journal.pone.0073829 PMC3743794

[17] Wallich C (1901) A method of recording egg development, for use of fish-culturists. Report of Commissioner of Fish and Fisheries. 185–195 p.

[18] Hamel P, Magnan P, East P, Lapointe M, Laurendeau P (1997) Comparison of different models to predict the in situ embryonic developmental rate of fish, with special reference to white sucker (*Catostomus commersoni*). Can J Fish Aquatic Sci 54(1):190–197.

[19] Alderdice DF, Velsen FPJ (1978) Relation between temperature and incubation time for eggs of chinook salmon (*Oncorhynchus tshawytscha*). Journal of the Fisheries Research Board of Canada 35:65–79.

[20] Konstantinov AS, Zdanovich VV (1986) Peculiarities of fish growth in relation to temperature fluctuation. J Ichthy 26(4):65–74.

[21] Blaxter JHS (1992) The effect of temperature on larval fishes. Netherlands J Zool 42(2–3):336–357.

[22] Stevenson JH (1965) Observations on grass carp in Arkansas: Prog Fish-Culturist 27(4):203–206.

[23] Galloway ML, Kilambi RV (1984) Temperature preference and tolerance of grass carp (*Ctenopharyngodon idella*). Arkansas Acad Sci Proc 38:36–37.

[24] Bettoli PW, Neill WH, Welsch SW (1985) Temperature preference and heat resistance of grass carp, *Ctenopharyngodon idella* (Valenciennes), bighead carp, *Hypophthalmichthys nobilis* (Gray), and their F_1_ hybrid. J Fish Bio 27:239–247.

[25] Chilton EW, Muoneke MI (1992) Biology and management of grass carp (*Ctenopharyngodon idella*, Cyprinidae) for vegetation control: a North American perspective: Rev Fish Bio Fisheries 2:283–320.

[26] Shireman JV, Smith CR (1983) Synopsis of biological data on the grass carp *Ctenopharyngodon idella* (Cuvier and Valenciennes, 1844) Rome, Italy: Food and Agriculture Organization of the United Nations Fisheries Synopsis 135. 86p.

[27] Hassler TJ (1970) Environmental influences on early development and year-class strength of northern pike in lakes Oahe and Sharpe, South Dakota: Trans Am Fish Soc 99(2):369–375.

[28] Hamel P, Magnan P, Lapointe M, East P (1997), Timing of spawning and assessment of a degree-day model to predict the in situ embryonic developmental rate of white sucker, *Catostomus commersoni* . Can J Fish Aquatic Sci 54:2040–2048.

[29] Geffen AJ, Nash RDM (2012) Egg development rates for use in egg production methods (EPMs) and beyond. Fish Res 117–118:48–62.

[30] Abdusamadov AS (1987) Biology of white amur, Ctenopharyngodon idella silver carp, Hypophthalmichthys molitrix, and bighead, Aristichthys nobilis, acclimatized in the Terek Region of the Caspian Basin. J Ichthy 26(3):41–49. 3657051

[31] Soin SG, Sukhanova AI (1972) Comparative morphological analysis of the development of the grass carp, the black carp, the silver carp and the bighead (Cyprinidae). J Ichthy 12(1):67–71.

[32] Fuiman LA, Trojnar JR (1980) Factors affecting egg diameter of white suckers (*Catostomus commersoni*). Copeia 1980(4): 699–704.

[33] Wu H, Tan J (2000) Relationship between egg sizes and body weight of parent fish of silver and bighead carps. Inland Water Fisheries 3:7–8.

[34] Chapman DC, Deters JE (2009) Effect of water hardness and dissolved-solid concentration on hatching success and egg size in bighead carp. Trans Am Fish Soc 138:1226–1231.

[35] Kawamura G, Shinoda Y (1980) Change in phototactic behaviour with growth of milkfish, *Chanos chanos* (Forsskal). Memoirs of the Kagoshima University Research Center for the South Pacific 1(1):75–87.

[36] Bulkowski L, Meade JW (1983) Changes in phototaxis during early development of walleye. Trans Am Fish Soc 112(3):445–447.

[37] Conner JV, Gallagher RP, Chatry MF (1980) Larval evidence for the natural reproduction of the grass carp (*Ctenophayngdon idella*) in the lower Mississippi River, in Fourth Annual Larval Fish Conference, Oxford, Mississippi, p. 1–19.

[38] Brown DJ, Coon TG (1991) Grass carp larvae in the lower Missouri River and its tributaries. North American Journal of Fisheries Management 11(1):62–66.

[39] Fuiman LA, Ottey DR (1993) Temperature effects on spontaneous behavior of larval and juvenile red drum *Sciaenops ocellatus*, and implications for foraging. Fish Bull 91:23–35.

[40] Batty RS (1994) The effect of temperature on the vertical distribution of larval herring (*Clupea harengus* L.). J Exp Mar Bio Eco 177:269–276.

[41] Green BS, Fisher R (2004) Temperature influences swimming speed, growth and larval duration in coral reef fish larvae. J Exp Mar Bio Eco 299:115–132.

[42] Knighton D (1998) Fluvial forms and processes: a new perspective John Wiley and Sons, Inc., New York.

[43] McDowall RM (2009) Early hatch: a strategy for safe downstream larval transport in amphidromous gobies. Rev Fish Bio Fisheries 19:1–8

[44] Inaba D, Nomura M, Nakamura M (1957) Preliminary report on the spawning of grass-carp and silver-carp in the Tone River, Japan and the development of their eggs. J Tokyo Univf Fisheries 43(1):81–101.

[45] Stanley JG, Miley WW, Sutton DL (1978) Reproductive requirements and likelihood for naturalization of escaped grass carp in the United States. Trans Am Fish Soc 107: 119–128.

[46] Kocovsky PM, Chapman DC, McKenna JE (2012) Thermal and hydrologic suitability of Lake Erie and its major tributaries for spawning of Asian carps. J Great Lakes Res 38:159–166.

[47] Wittmann ME, Jerde CL, Howeth JG, Maher SP, Deines AM, et al (2014) Grass carp in the Great Lakes region: establishment potential, expert perceptions, and re-evaluation of experimental evidence of ecological impact. Can J Fish Aquatic Sci 71: 992–999.

[48] Cudmore B, Mandrak NE (2004) Biological synopsis of grass carp (Ctenopharyngodon idella): Canadian Manuscript Report of Fisheries and Aquatic Sciences 2705, 44 pp.

[49] Cudmore B, Mandrak NE (2011) Assessing the biological risk of Asian carps to Canada, *in* Chapman D.C., and Hoff M.H. eds., Invasive Asian Carps in North America: American Fisheries Society Bethesda, Maryland, p.15–30.

[50] Hargrave CW, Gido KB (2004) Evidence of reproduction by exotic grass carp in the Red and Washita River, Oklahoma. Southwest Nat 49(1):89–93.

[51] Deters JE, Chapman DC, McElroy B (2013) Location and timing of Asian carp spawning in the lower Missouri River. Env Bio Fish 96:617–629.

