# Supplementary material for: Embryonic and Larval Development and Early Behavior in Grass Carp, Ctenopharyngodon idella: Implications for Recruitment in Rivers
Source: PLoS One. 2015 Mar 30;10(3):e0119023. doi: 10.1371/journal.pone.0119023 (PMC4378990; doi:10.1371/journal.pone.0119023)
Supplement: S1 File — (DOCX) [file pone.0119023.s001.docx]

Figure 1) Unfertilized eggs (Live, 10x).

Figure 2) 1-cell stage (Preserved, 20x).

Figure 3) 2-cell stage (Live, 10x; Preserved, 30x). Arrows indicate individual eggs at the two cell stage.

Figure 4) 4-cell stage (Preserved, 30x; Preserved, 30x), Line drawing from Yi and others (1988).

Figure 5) 8-cell stage (Live, 10x; Preserved, 40x), Line drawing from Yi and others (1988).

Figure 6) 16-cell stage (Preserved, 36x; preserved, 50x), Line drawing from Yi and others (1988).

Figure 7) 32-cell stage (Preserved, 50x; preserved 60x). Line drawing from Yi and others (1988).

Figure 8) 64-cell stage (Preserved, 50x; preserved 70x). Line drawing from Yi and others (1988).

Figure 9) 128-cell stage (Preserved, 50x; preserved 50x). Line drawing from Yi and others (1988).

Figure 10) Morula stage (Preserved, 50x; preserved, 60x). Line drawing from Yi and others (1988).

Figure 11) Early blastula stage (Preserved, 50x; preserved, 70x). Line drawing from Yi and others (1988).

Figure 12) Mid-blastula stage (Preserved, 40x; preserved, 40x). Line drawing from Yi and others (1988).

Figure 13) Late blastula stage (Preserved, 40x; preserved, 70x). Line drawing from Yi and others (1988).

Figure 14) Early gastrula stage (Preserved, 30x; preserved, 70x). Line drawing from Yi and others (1988).

Figure 15) Mid-gastrula stage (Preserved 30x; preserved 40x). Line drawing from Yi and others (1988).

Figure 16) Late gastrula stage (Preserved, 28x; preserved, 40x). Line drawing from Yi and others (1988).

Figure 17) Neurula stage (Preserved, 40x; preserved, 60x). Line drawing from Yi and others (1988).

Figure 18) Blastopore closure stage (Preserved, 40x; preserved 50x). Line drawing from Yi and others (1988).

Figure 19) Somite appearance stage (preserved, 40x; preserved, 50x). Line drawing from Yi and others (1988).

Figure 20) Optic primordium stage (preserved, 40x, 60x). Line drawing from Yi and others (1988). Arrow indicates the optic primordium.

Figure 21) Optic vesicle stage (Preserved, 30x; preserved,70x). Line drawing from Yi and others (1988).

Figure 22) Olfactory placode stage (Preserved, 30x; preserved, 50x). Line drawing from Yi and others (1988).

Figure 23) Tail bud stage (Preserved, 40x; preserved, 60x). Line drawing from Yi and others (1988).

Figure 24) Otic capsule stage (Preserved, 60x; preserved, 40x). Line drawing from Yi and others (1988). Arrow indicates position of the otic capsule.

Figure 25) Tail vesicle stage (Preserved, 50x; preserved, 50x). Line drawing from Yi and others (1988). Arrow indicates position of the tail vesicle.

Figure 26) Caudal fin stage (Preserved, 50x; preserved, 50x). Line drawing from Yi and others (1988). Arrow indicates position of the tail vesicle.

Figure 27) Lens formation stage (Preserved, 30x; preserved, 30x). Line drawing from Yi and others (1988).

Figure 28) Muscular effect stage (Preserved, 50x; preserved, 30x). Line drawing from Yi and others (1988).

Figure 29) Heart rudiment stage (Live, 10x; preserved, 30x). Line drawing from Yi and others (1988).

Figure 30) Otolith appearance stage (preserved, 112.5x; preserved, 4x). Arrows point to otoliths. Line drawing from Yi and others (1988).

Figure 31) Heartbeat stage (Live, 30x; preserved, 30x). Line drawing from Yi and others (1988).

Figure 32) Hatching stage (preserved, 30x; preserved, 20x). Note lack of eyespots. Line drawing from Yi and others (1988).

Figure 33) Rudimentary pectoral fin stage (Preserved, 20x; preserved, 70x). Line drawing from Yi and others (1988).

Figure 34) Gill arch stage (Live, 10x; preserved, 70x). Line drawing from Yi and others (1988).

Figure 35) Xanthic eye stage (Live, 20x; preserved, 20x). Line drawing from Yi and others (1988).

Figure 36) Gill filament stage (preserved, 20x; preserved, 80x). Line drawing from Yi and others (1988).

Figure 37) Melanoid eye stage (preserved, 40x, stage incomplete; preserved, 20x, stage complete). Line drawing from Yi and others (1988).

Figure 38) Gas bladder emergence stage (live, 40x; preserved, 20x). Line drawing from Yi and others (1988).

Figure 39) One chamber gas bladder stage (live, 10x; preserved, 20x). Line drawing from Yi and others (1988).

Figure 40) Yolk sac absorption stage (Live, 10x; preserved, 20x).

Figure 41) (Top to bottom) Comparison of grass carp, bighead carp, and silver carp larvae, at developmental stage 32-33 (preserved, 20x).

Figures


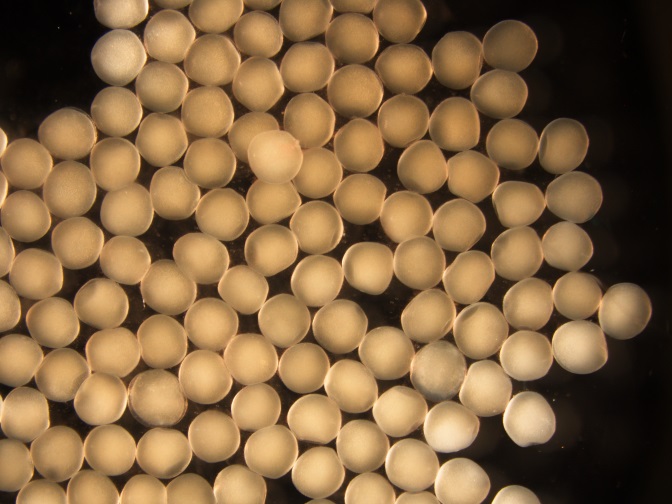


Figure 1) Unfertilized eggs (Live, 10x).


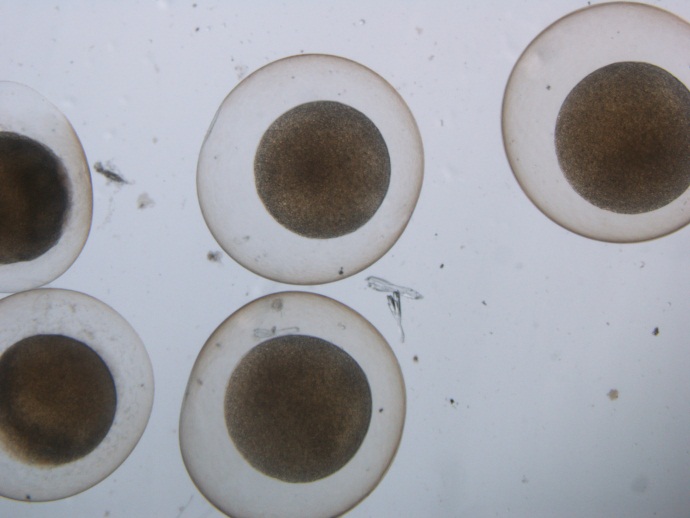


Figure 2) 1-cell stage (Preserved, 20x).


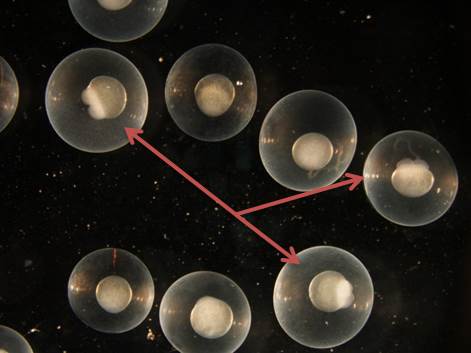

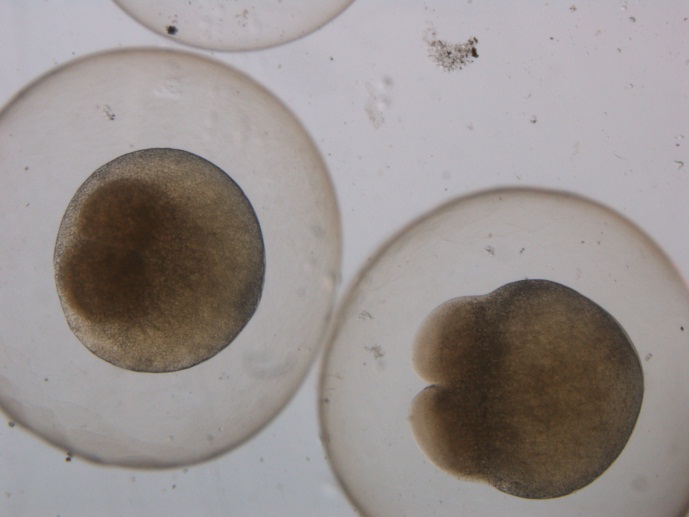


Figure 3) 2-cell stage (Live, 10x; Preserved, 30x). Arrows indicate individual eggs at the two cell stage.


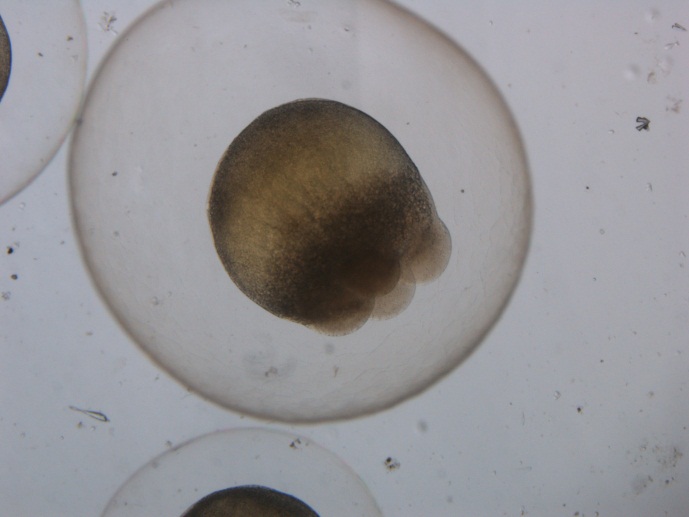

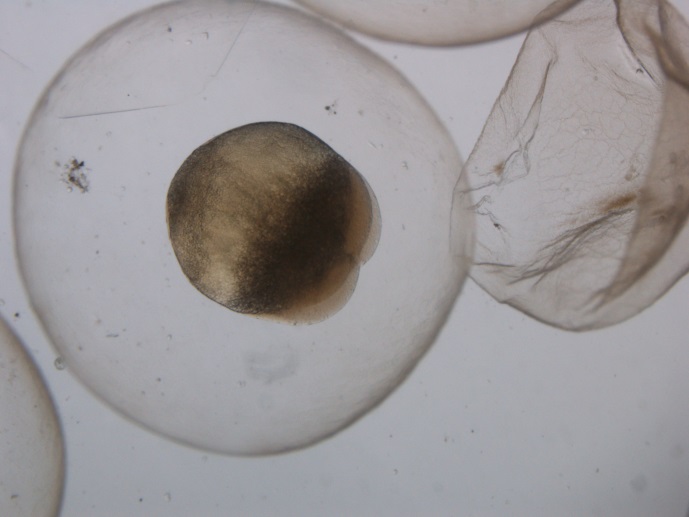

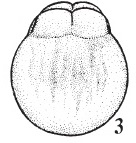


Figure 4) 4-cell stage (Preserved, 30x; Preserved, 30x), Line drawing from Yi and others (1988).


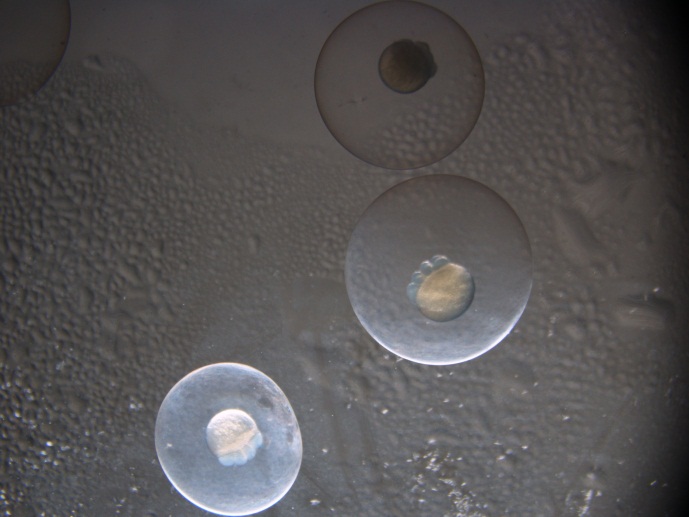

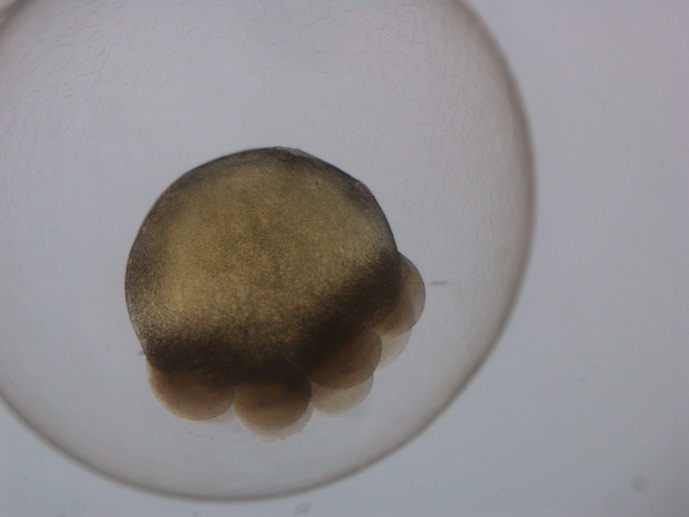

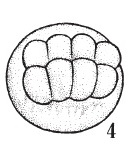


Figure 5) 8-cell stage (Live, 10x; Preserved, 40x), Line drawing from Yi and others (1988).


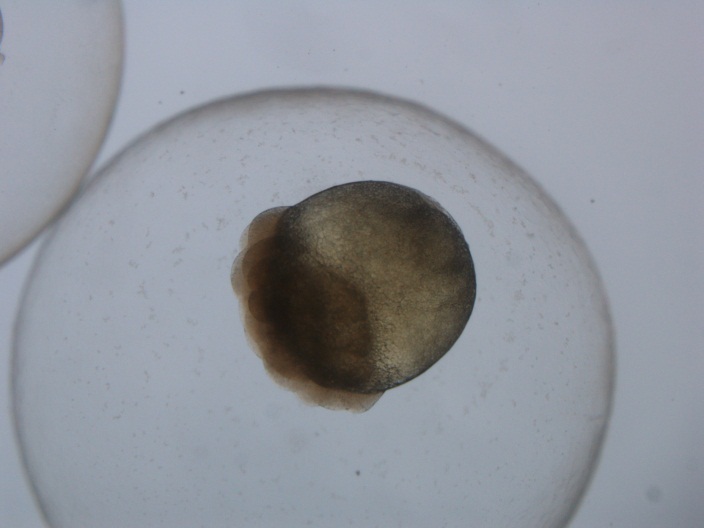

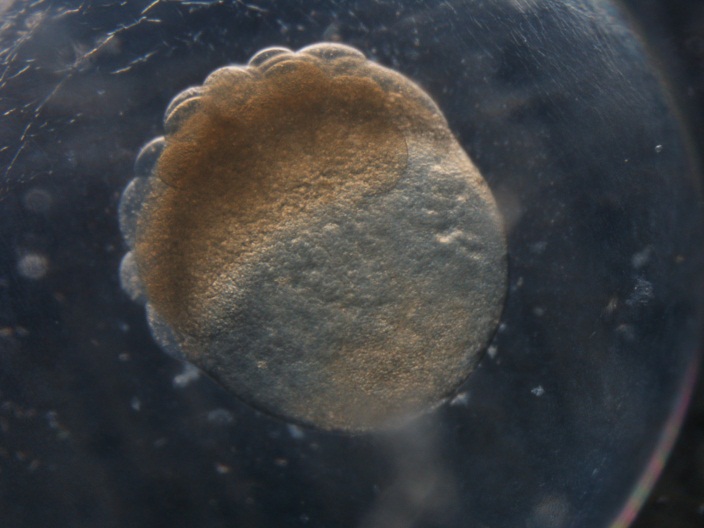

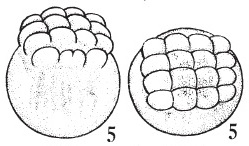


Figure 6) 16-cell stage (Preserved, 36x; preserved, 50x), Line drawing from Yi and others (1988).


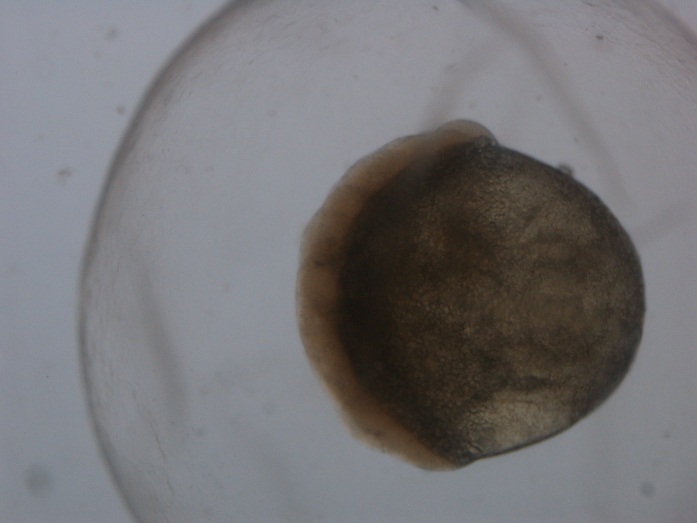

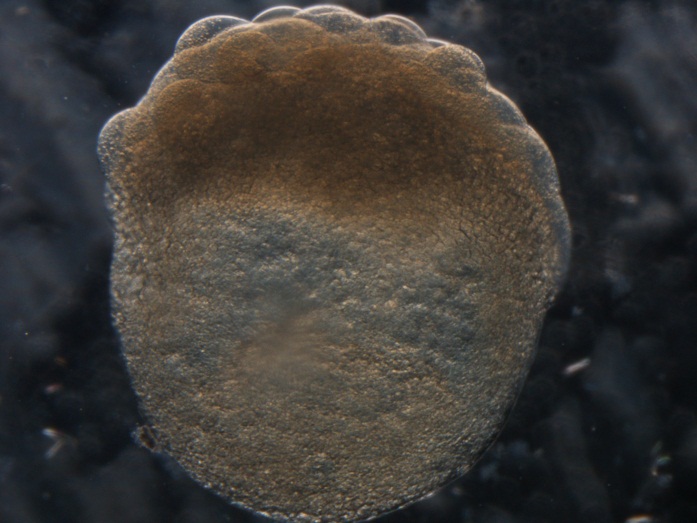

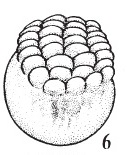


Figure 7) 32-cell stage (Preserved, 50x; preserved 60x). Line drawing from Yi and others (1988).


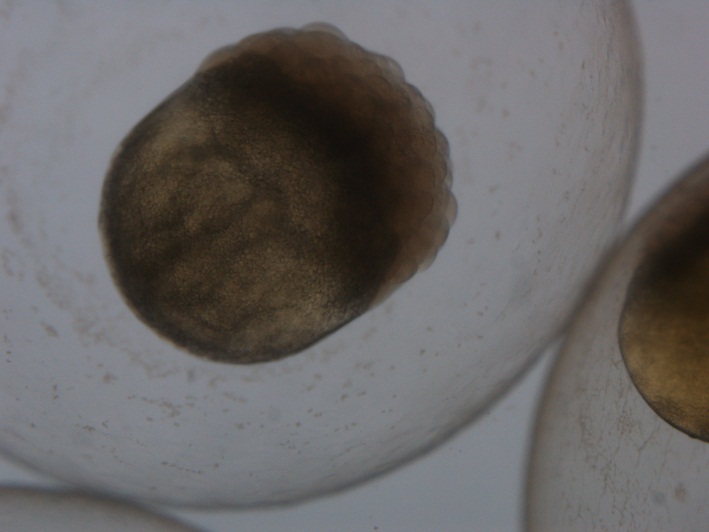

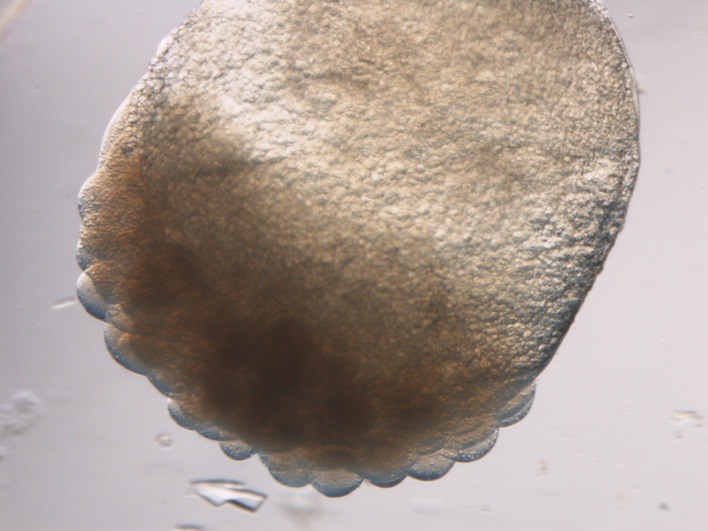

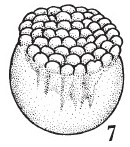


Figure 8) 64-cell stage (Preserved, 50x; preserved 70x). Line drawing from Yi and others (1988).


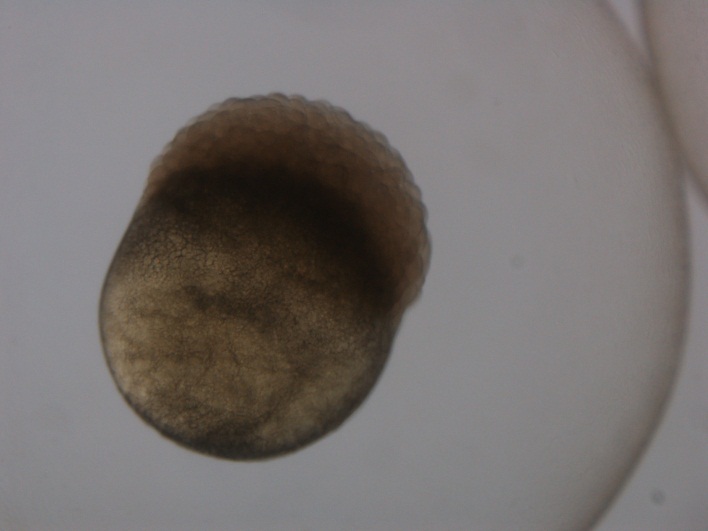

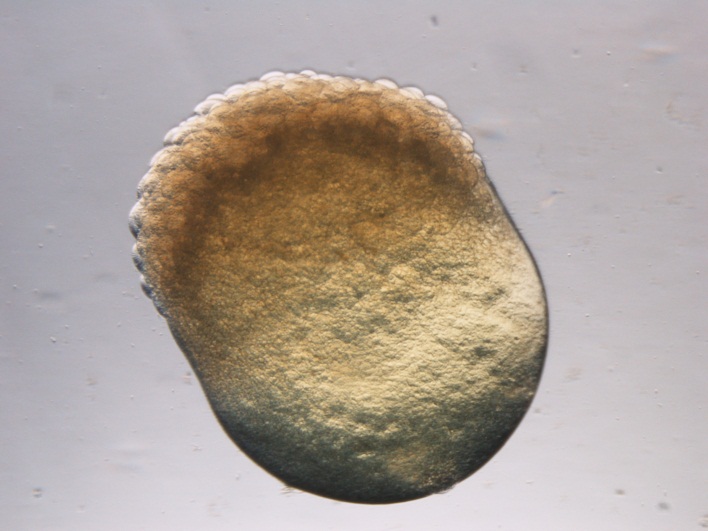

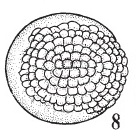


Figure 9) 128-cell stage (Preserved, 50x; preserved 50x). Line drawing from Yi and others (1988).


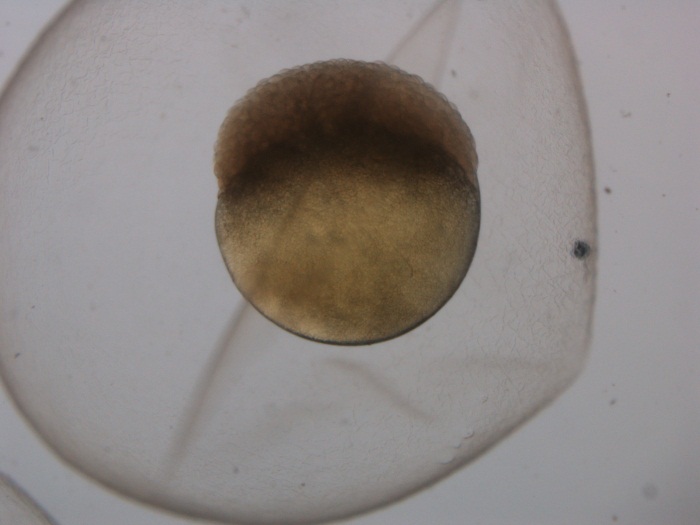

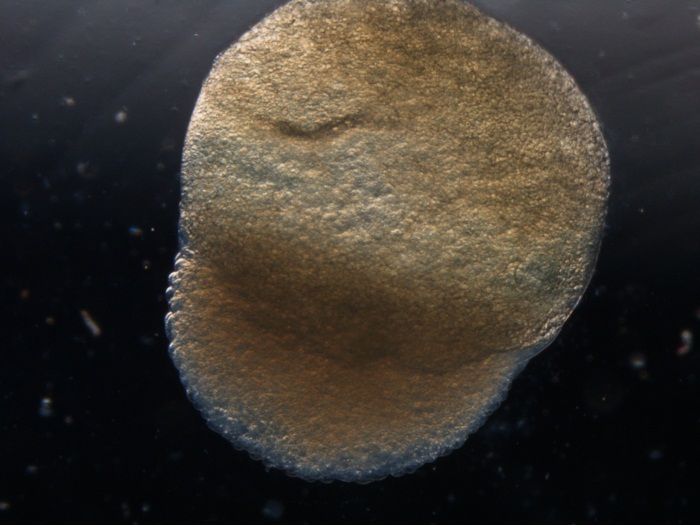

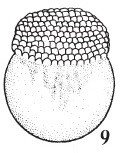


Figure 10) Morula stage (Preserved, 50x; preserved, 60x). Line drawing from Yi and others (1988).


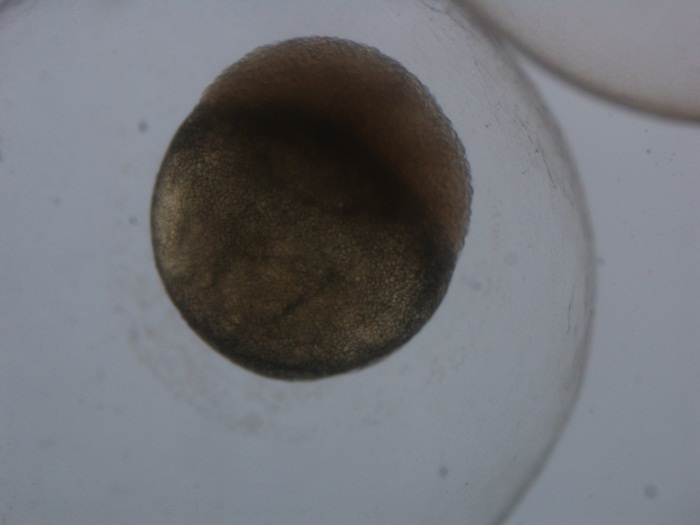

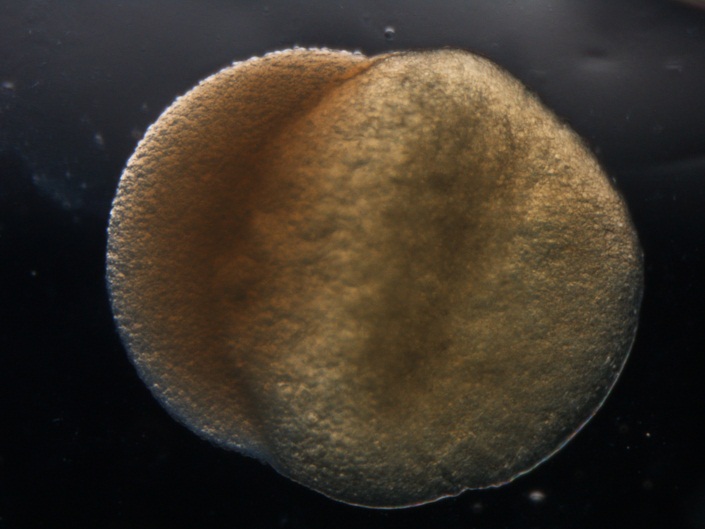

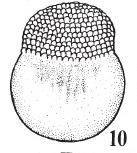


Figure 11) Early blastula stage (Preserved, 50x; preserved, 70x). Line drawing from Yi and others (1988).


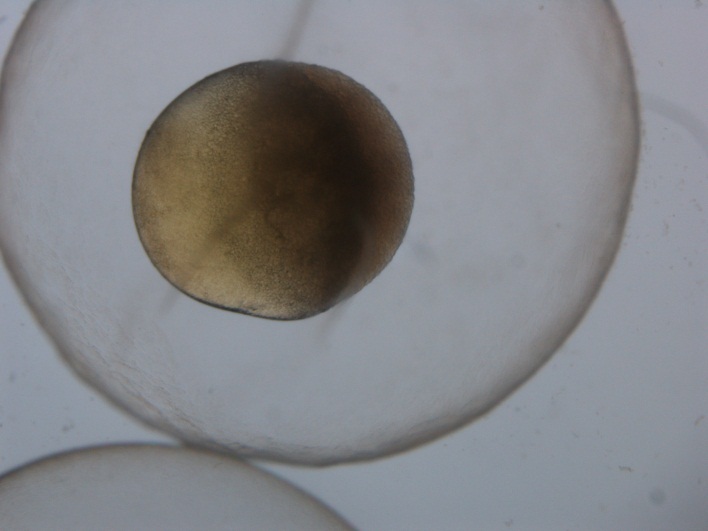

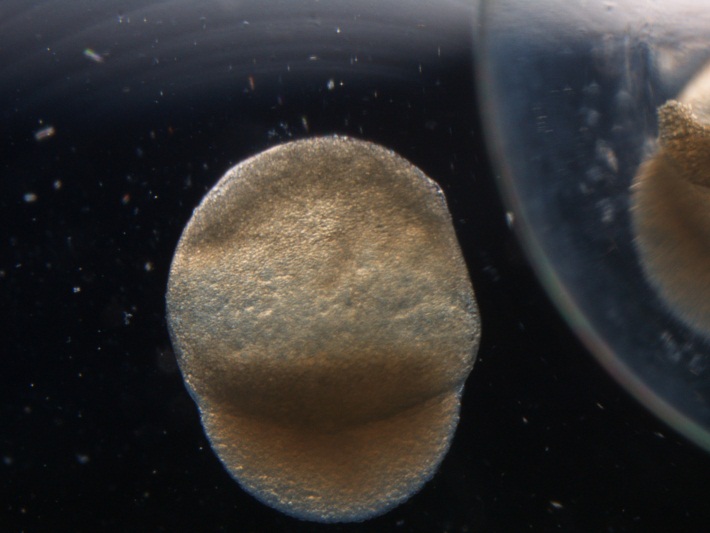

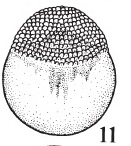


Figure 12) Mid-blastula stage (Preserved, 40x; preserved, 40x). Line drawing from Yi and others (1988).


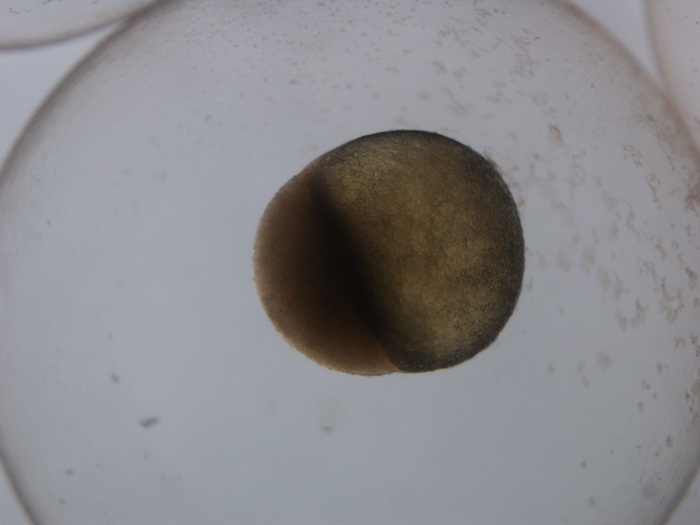

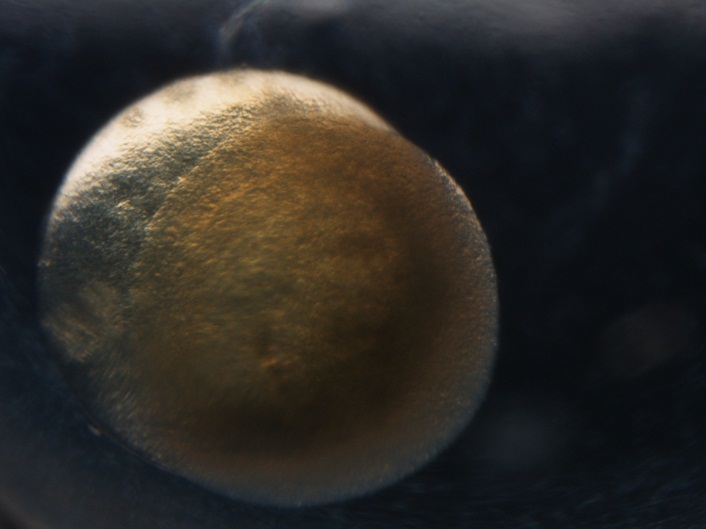

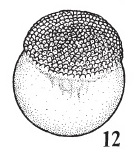


Figure 13) Late blastula stage (Preserved, 40x; preserved, 70x). Line drawing from Yi and others (1988).


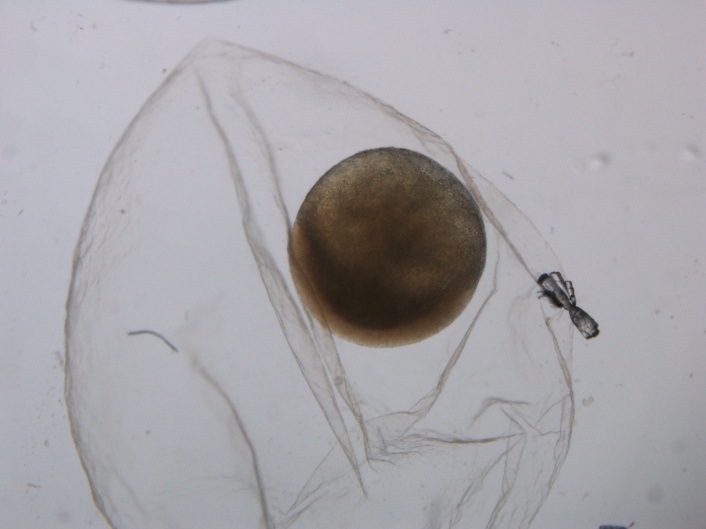

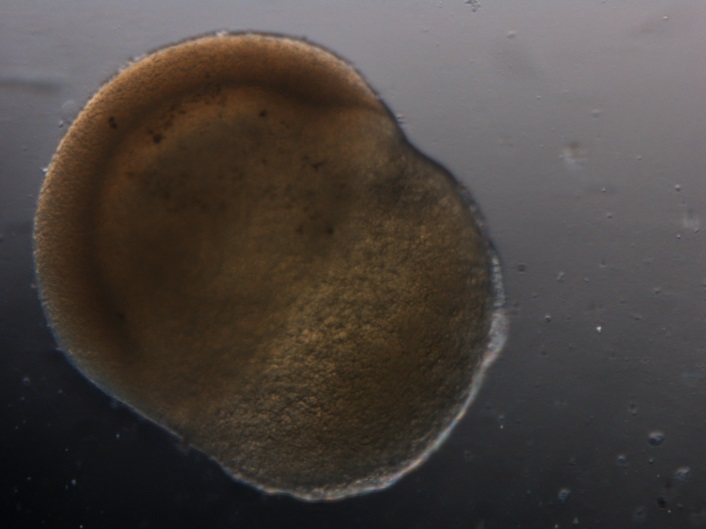

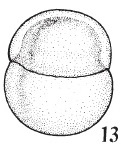


Figure 14) Early gastrula stage (Preserved, 30x; preserved, 70x). Line drawing from Yi and others (1988).


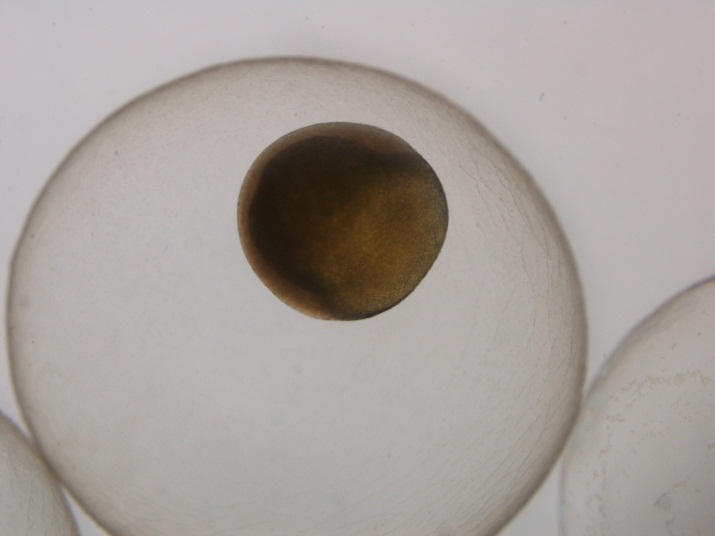

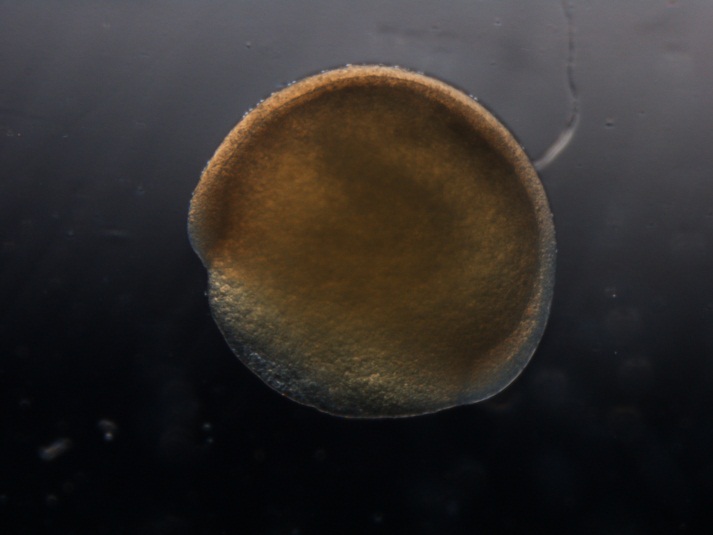

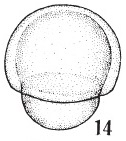


Figure 15) Mid-gastrula stage (Preserved 30x; preserved 40x). Line drawing from Yi and others (1988).


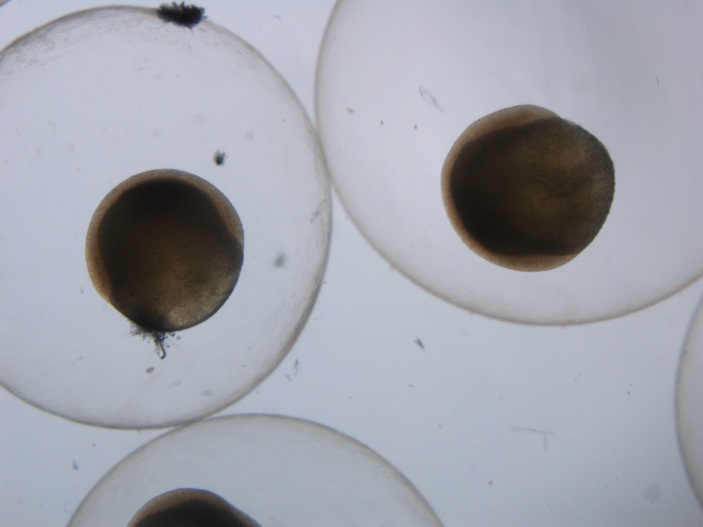

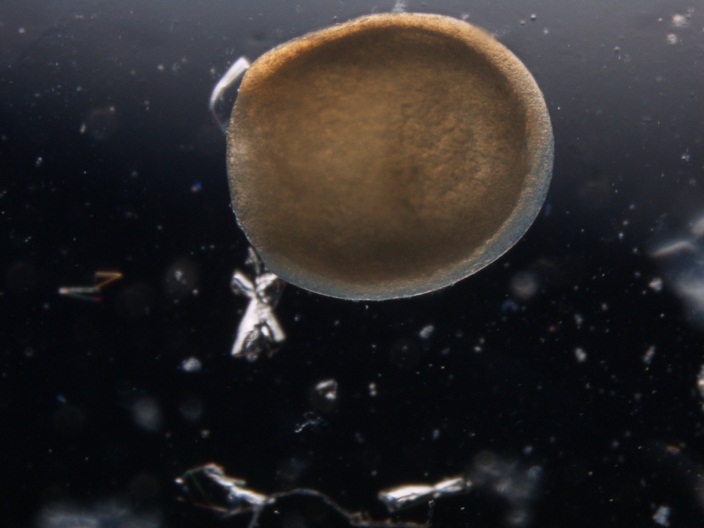

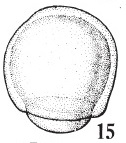


Figure 16) Late gastrula stage (Preserved, 28x; preserved, 40x). Line drawing from Yi and others (1988).


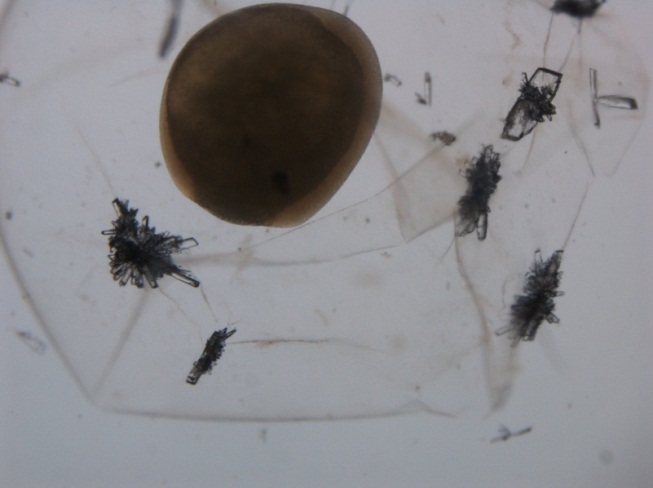

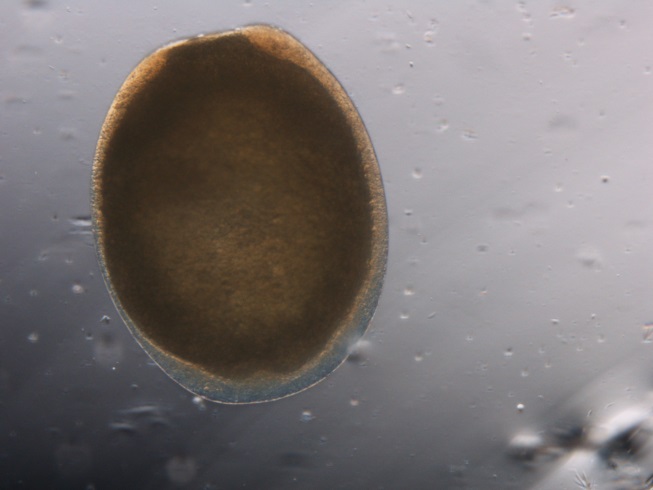

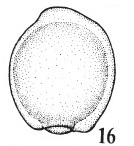


Figure 17) Neurula stage (Preserved, 40x; preserved, 60x). Line drawing from Yi and others (1988).


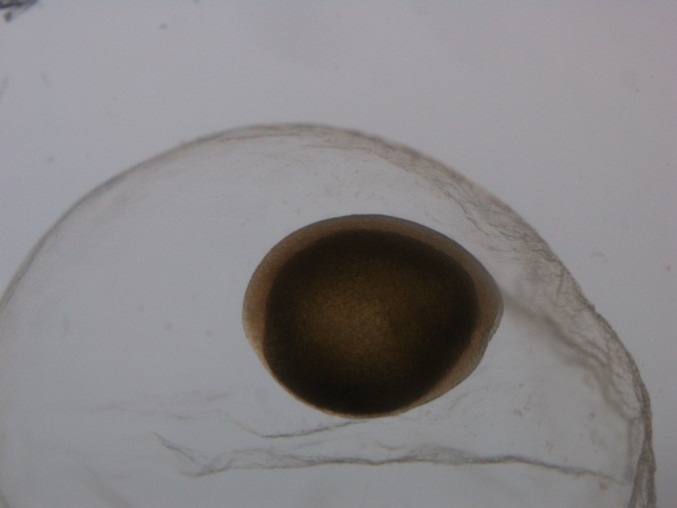

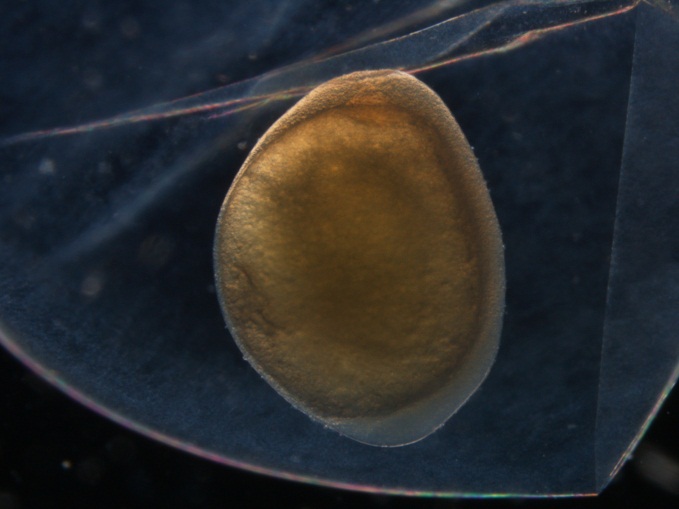

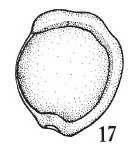


Figure 18) Blastopore closure stage (Preserved, 40x; preserved 50x). Line drawing from Yi and others (1988).


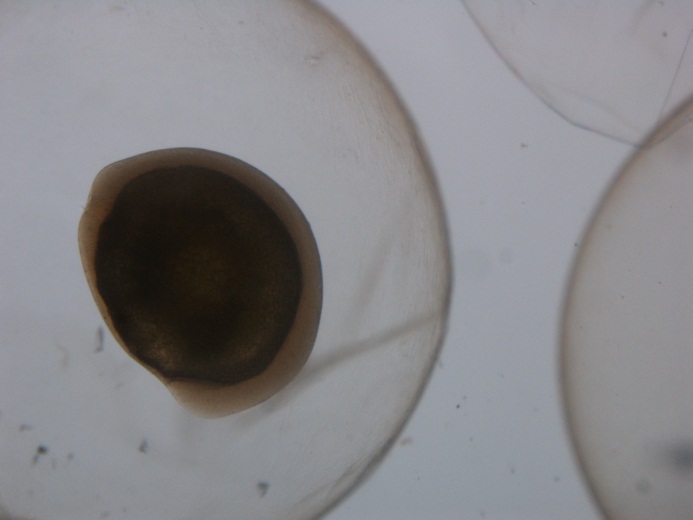

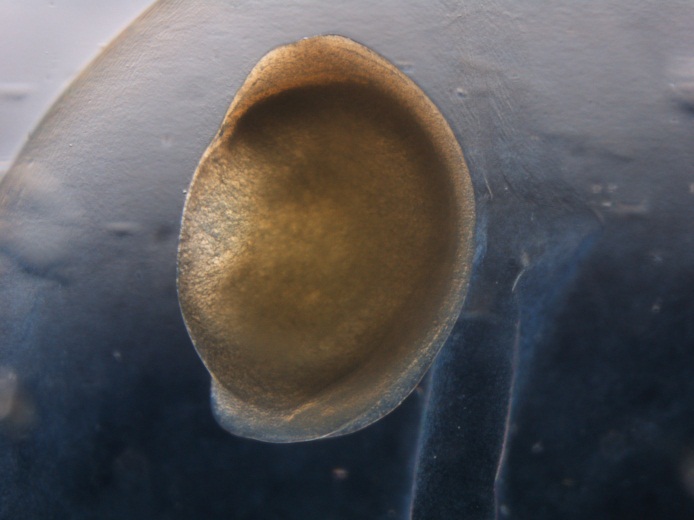

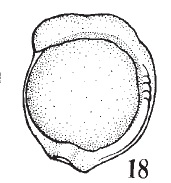


Figure 19) Somite appearance stage (preserved, 40x; preserved, 50x). Line drawing from Yi and others (1988).


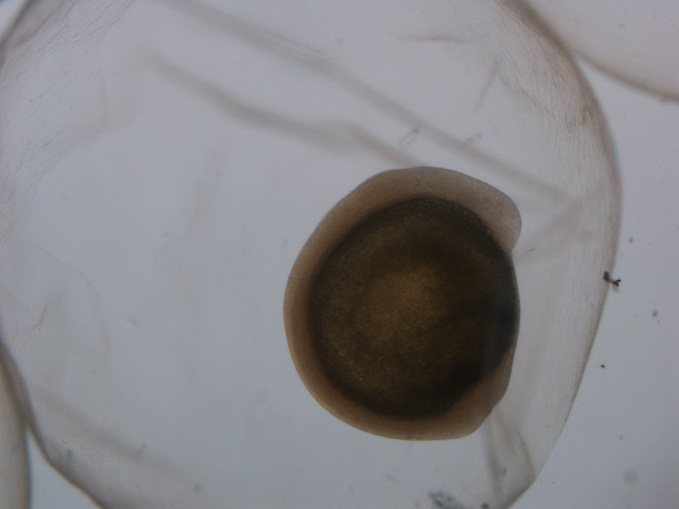

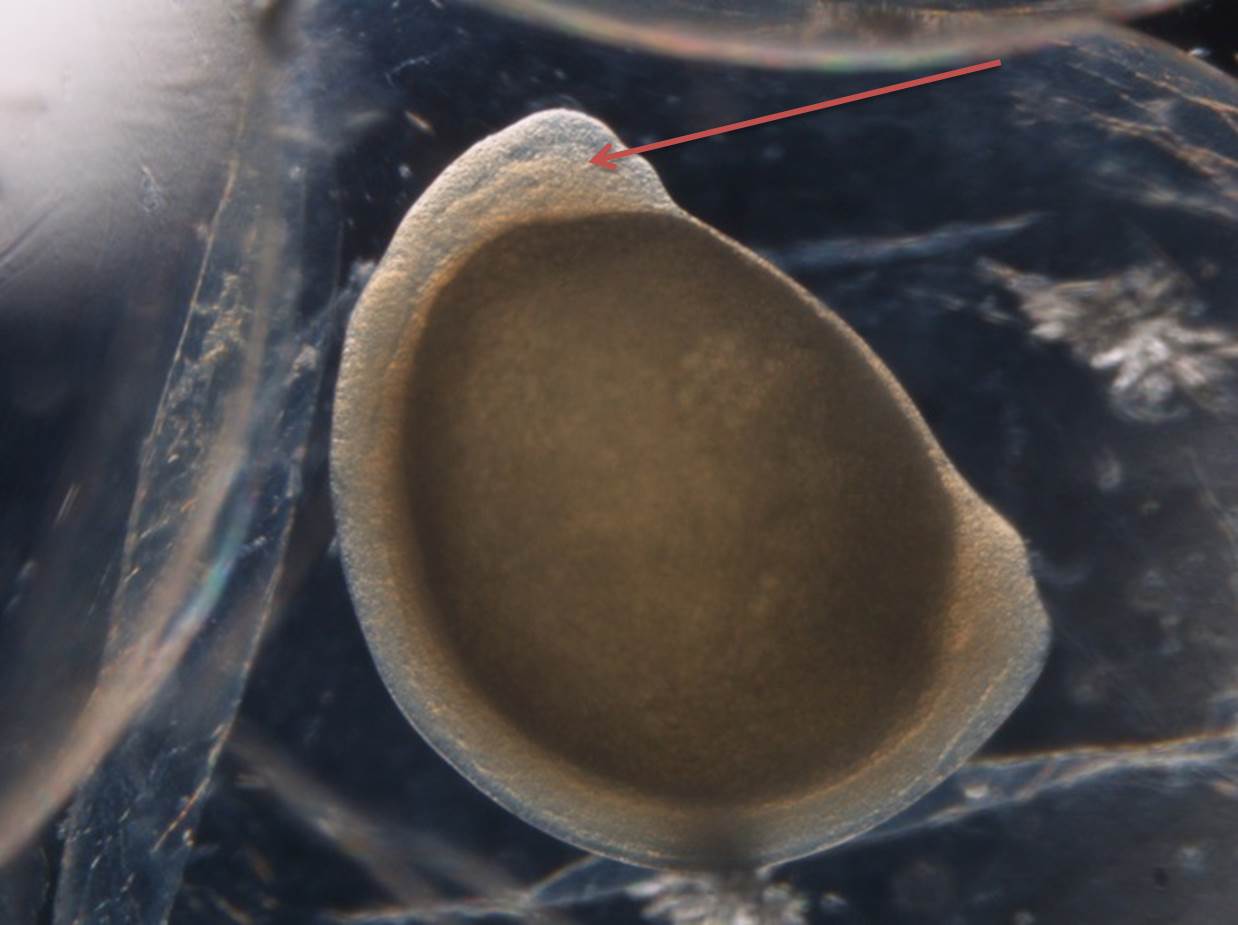

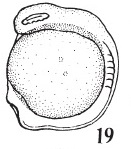


Figure 20) Optic primordium stage (preserved, 40x, 60x). Line drawing from Yi and others (1988). Arrow indicates the optic primordium.


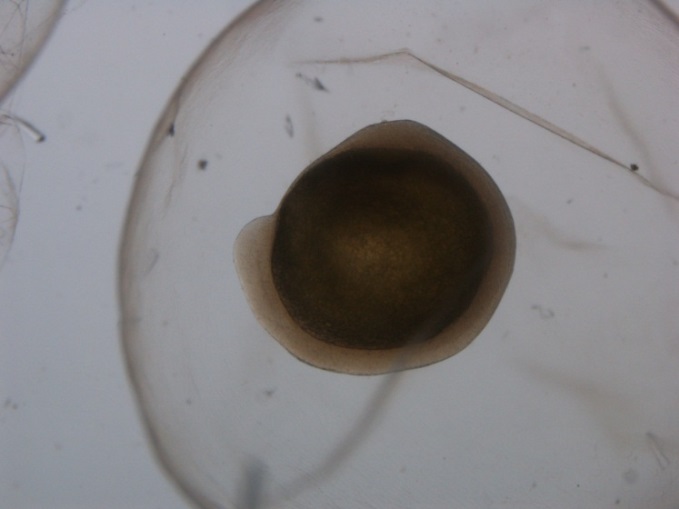

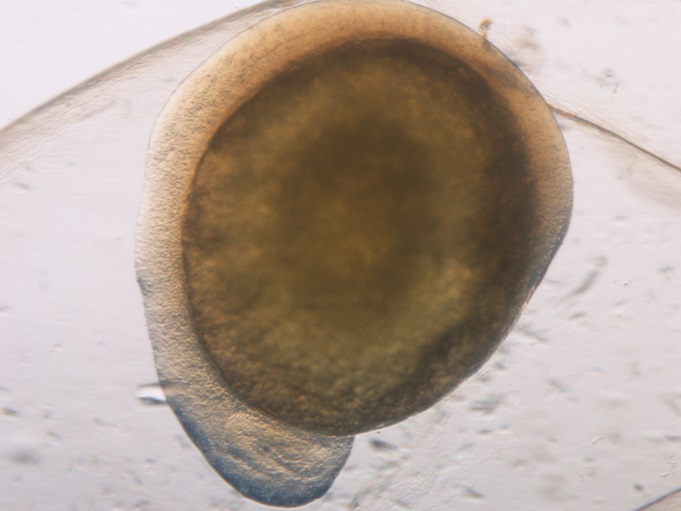

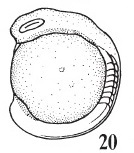


Figure 21) Optic vesicle stage (Preserved, 30x; preserved 70x). Line drawing from Yi and others (1988).


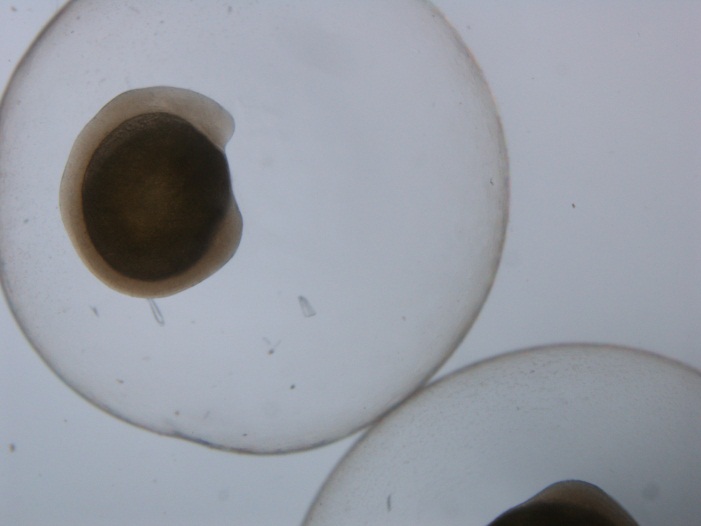

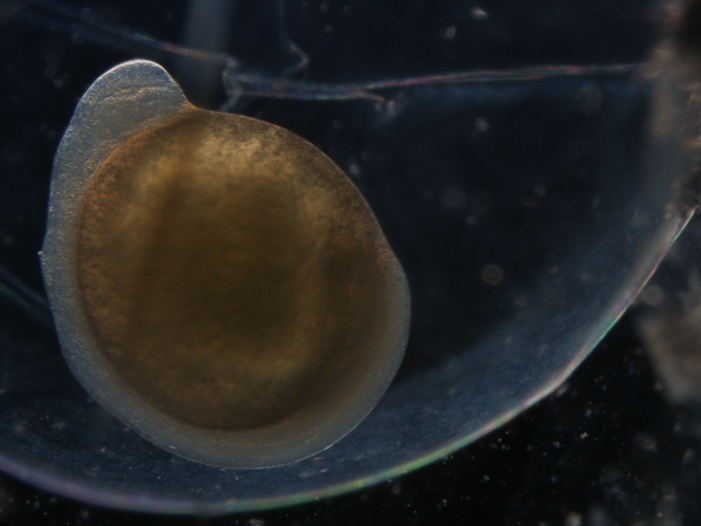

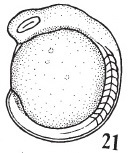


Figure 22) Olfactory placode stage (Preserved, 30x; preserved 50x). Line drawing from Yi and others (1988).


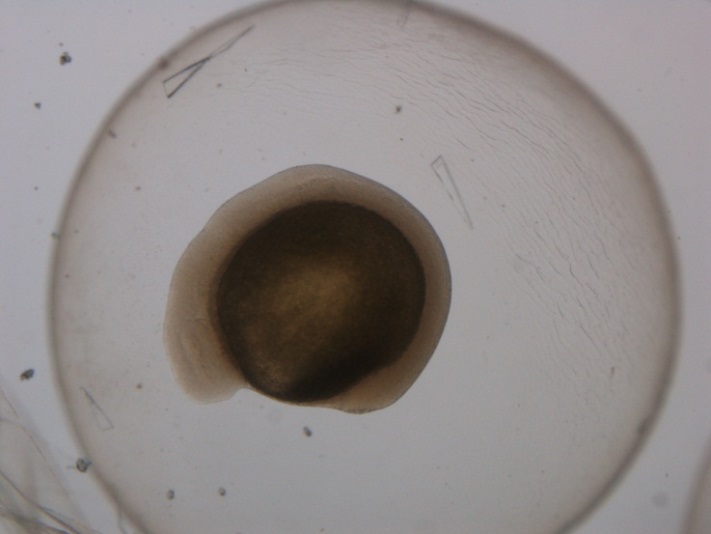

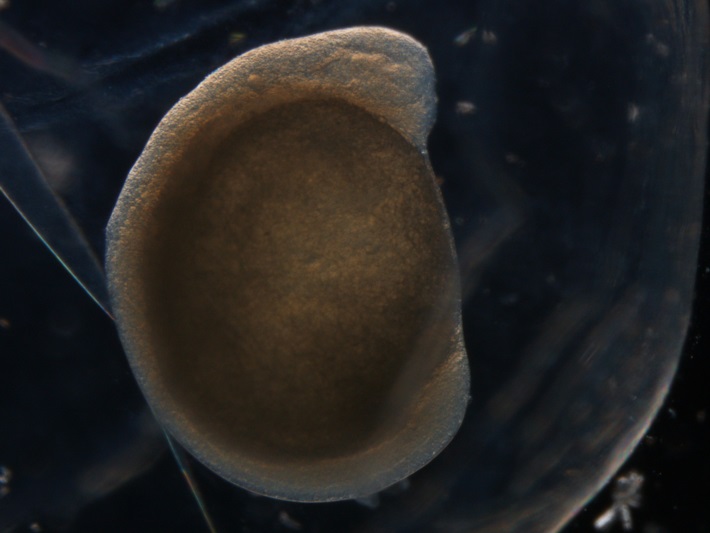

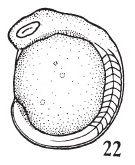


Figure 23) Tail bud stage (Preserved, 40x; preserved, 60x). Line drawing from Yi and others (1988).


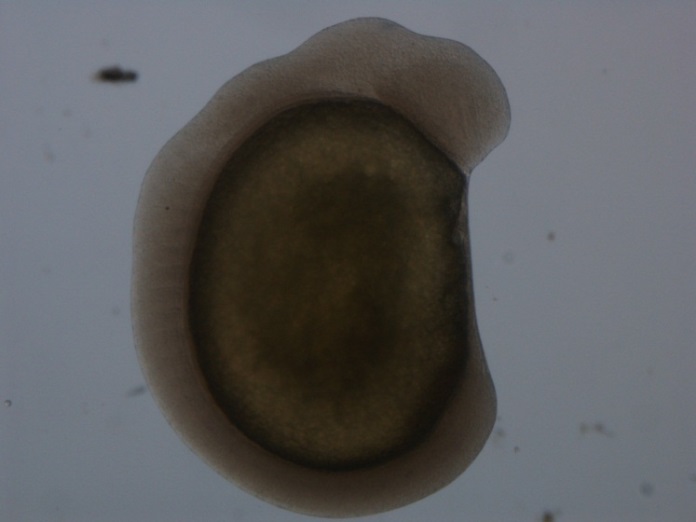

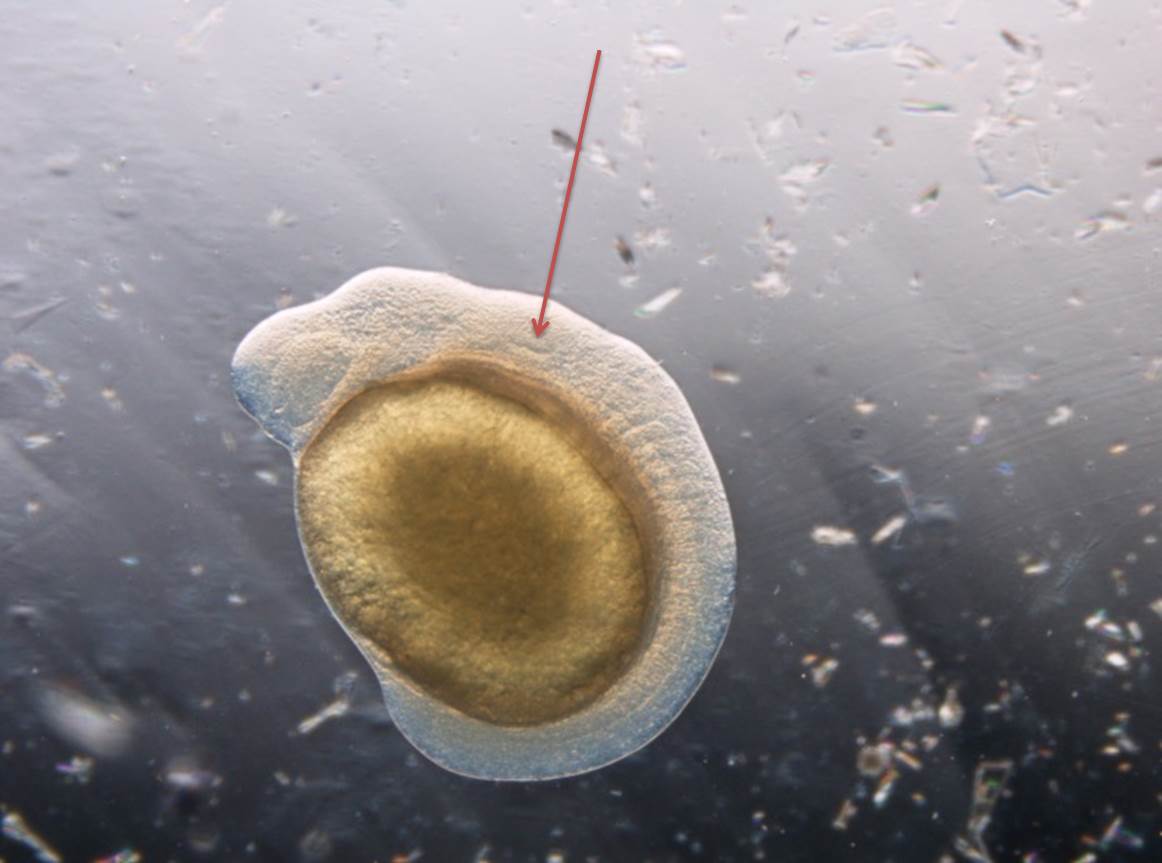

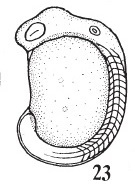


Figure 24) Otic capsule stage (Preserved, 60x; preserved, 40x). Line drawing from Yi and others (1988). Arrow indicates position of the otic capsule.


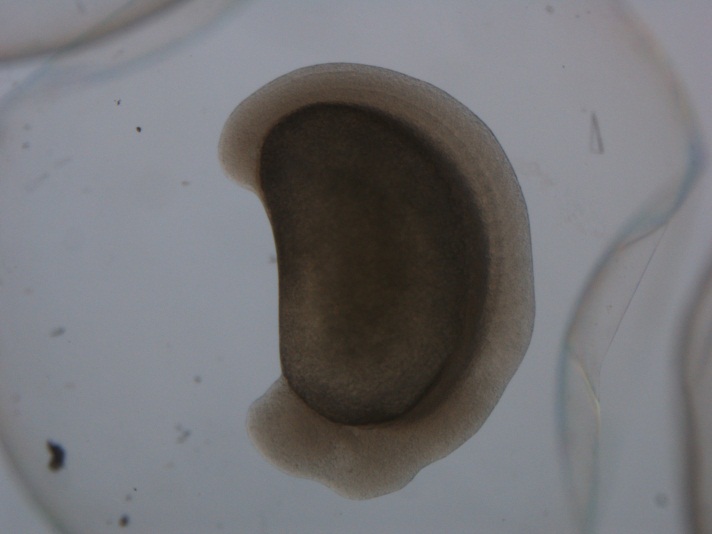

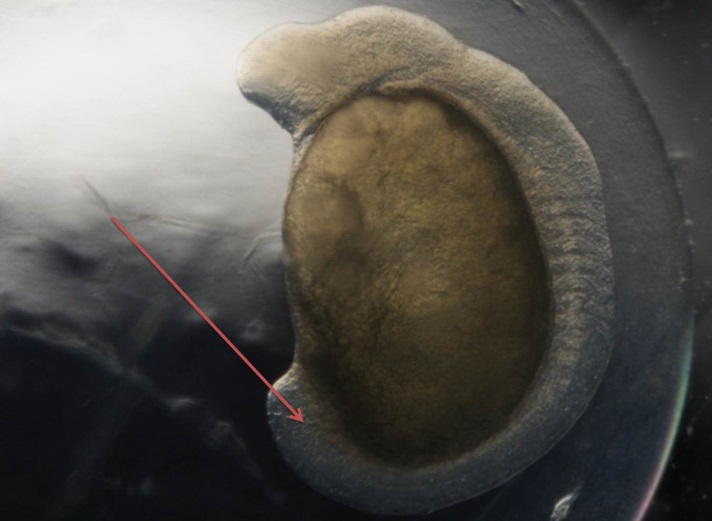

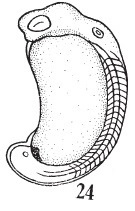


Figure 25) Tail vesicle stage (Preserved, 50x; preserved, 50x). Line drawing from Yi and others (1988). Arrow indicates position of the tail vesicle.


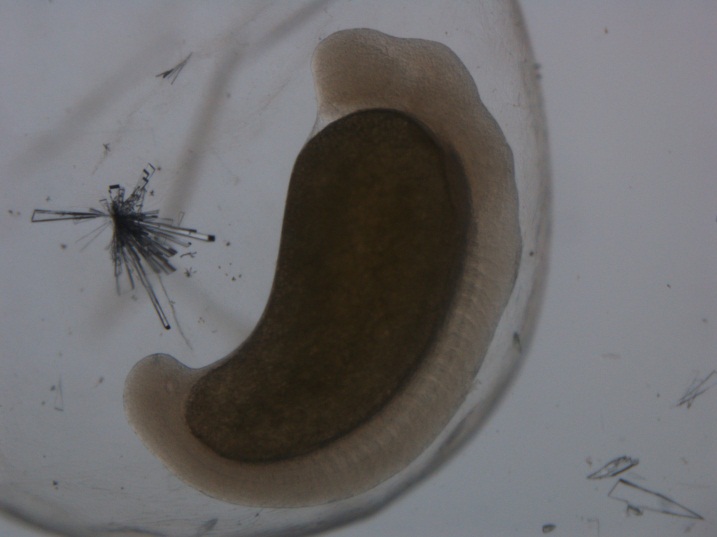

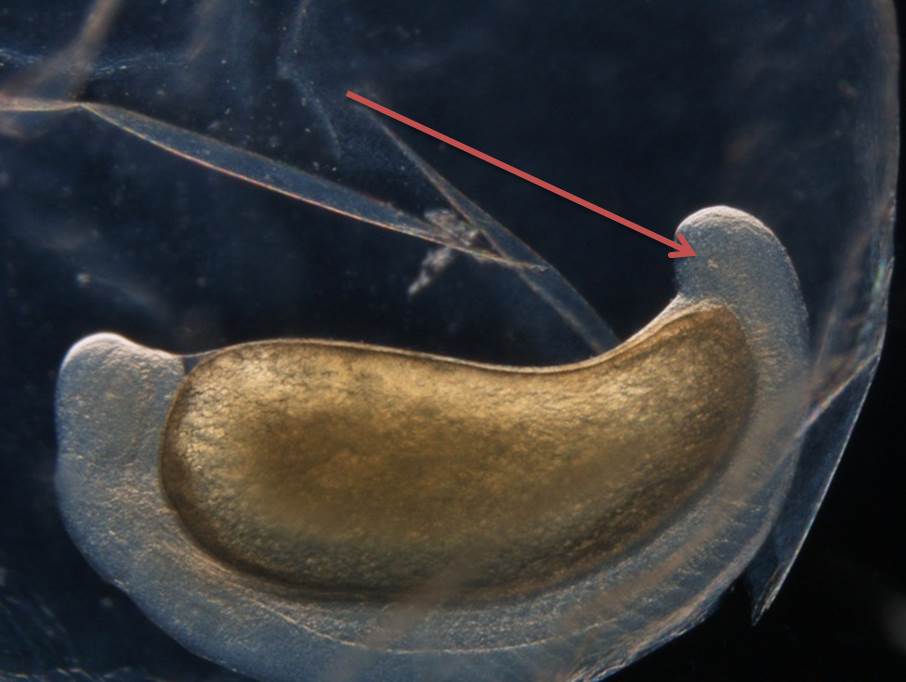

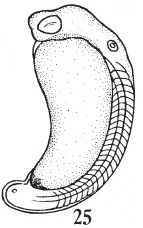


Figure 26) Caudal fin stage (Preserved, 50x; preserved, 50x). Line drawing from Yi and others (1988). Arrow indicates position of the tail vesicle.


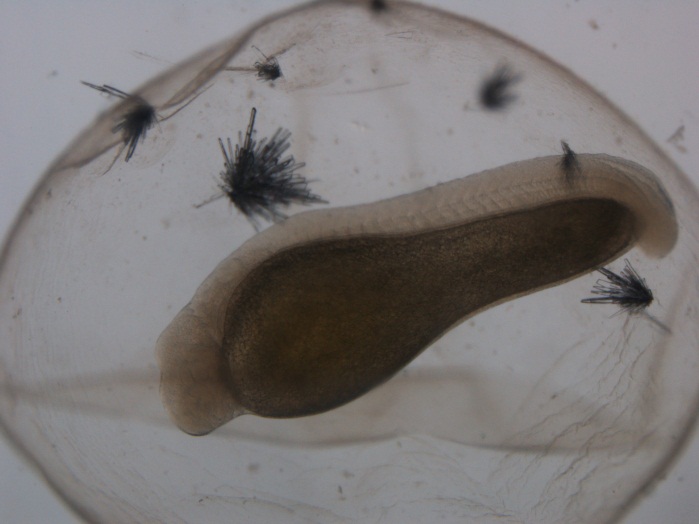

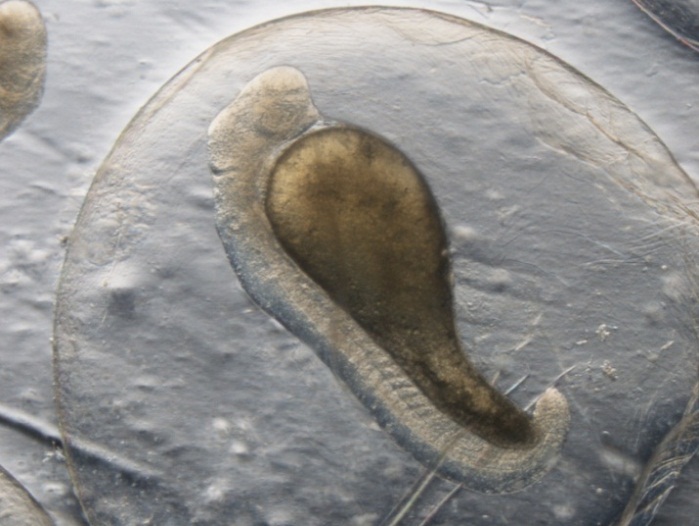


Figure 27) Lens formation stage (Preserved, 30x; preserved, 30x). Line drawing from Yi and others (1988).


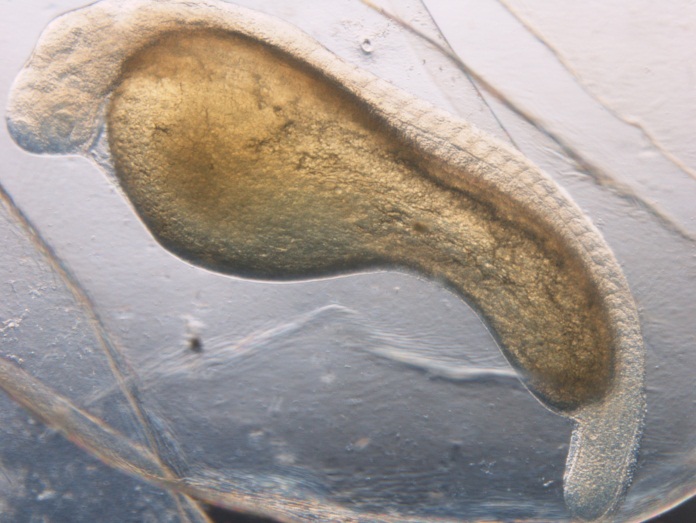

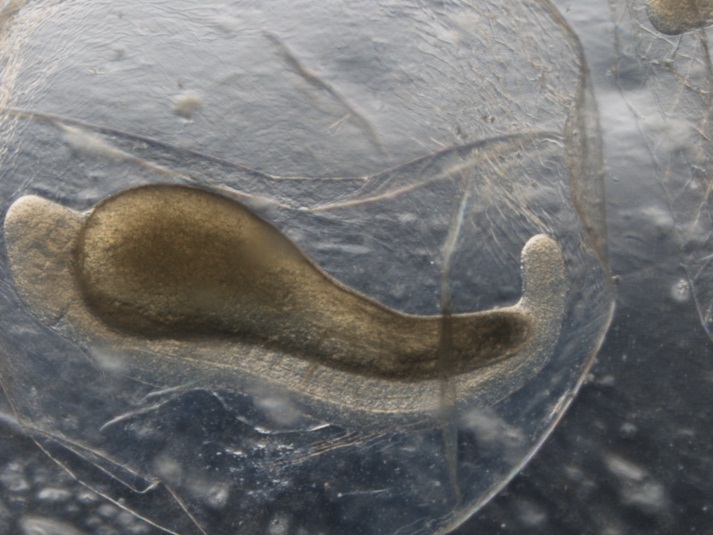

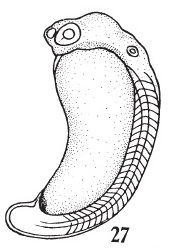


Figure 28) Muscular effect stage (Preserved, 50x; preserved, 30x). Line drawing from Yi and others (1988).


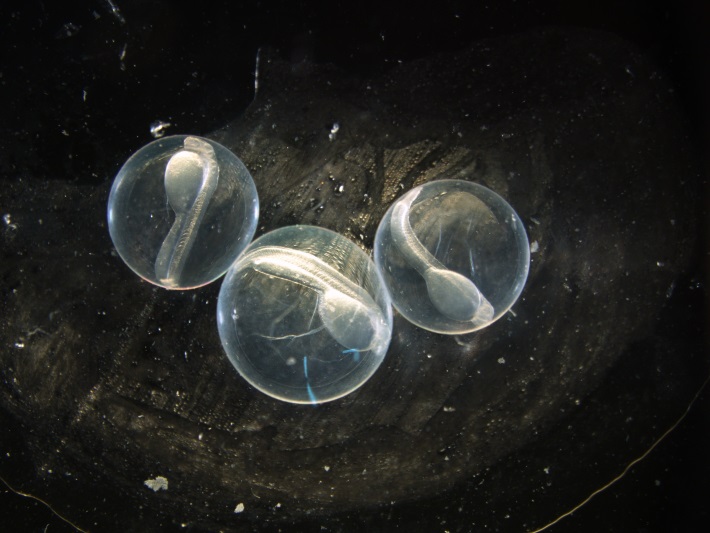

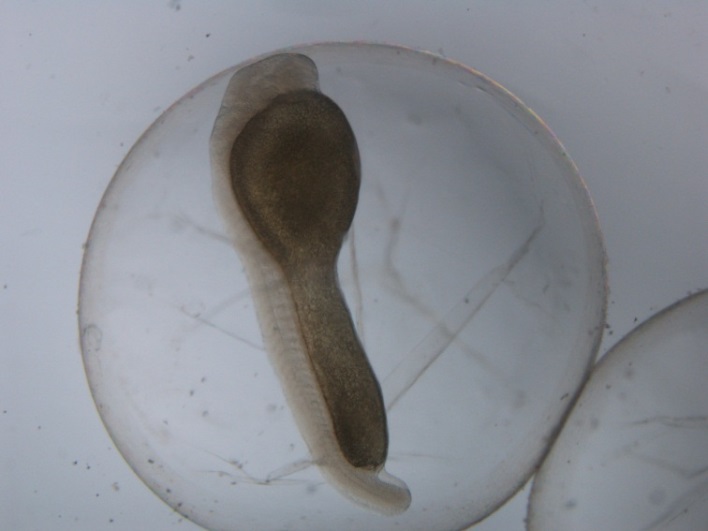

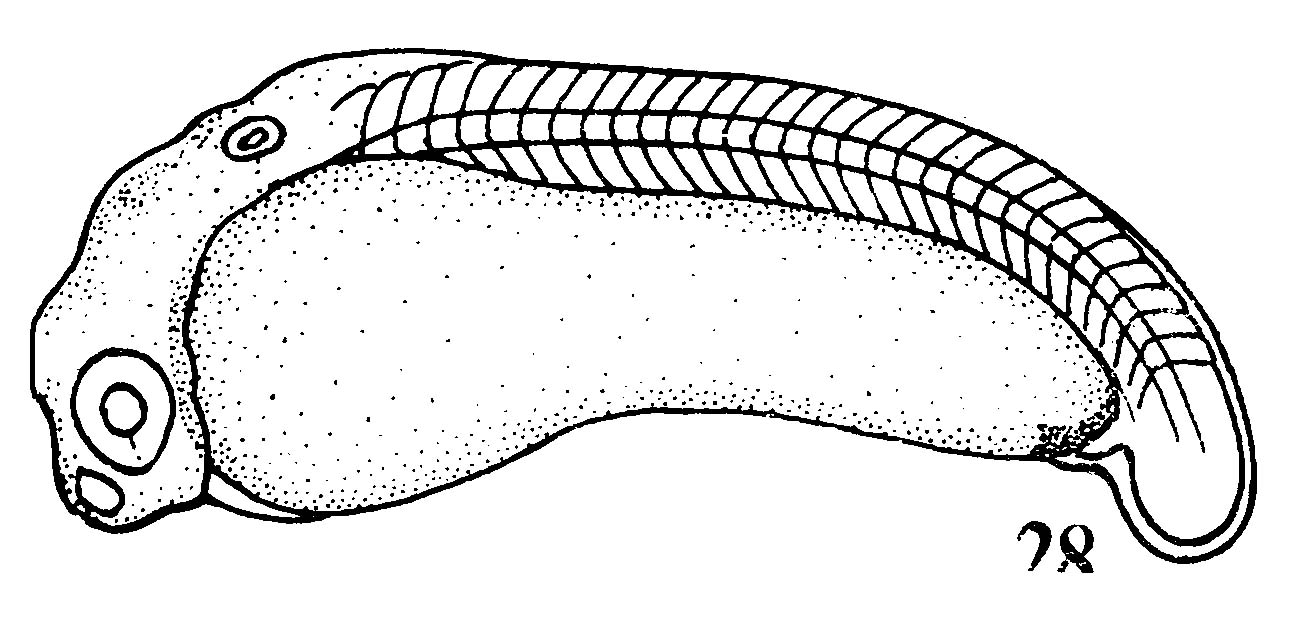


Figure 29) Heart rudiment stage (Live, 10x; preserved, 30x). Line drawing from Yi and others (1988).


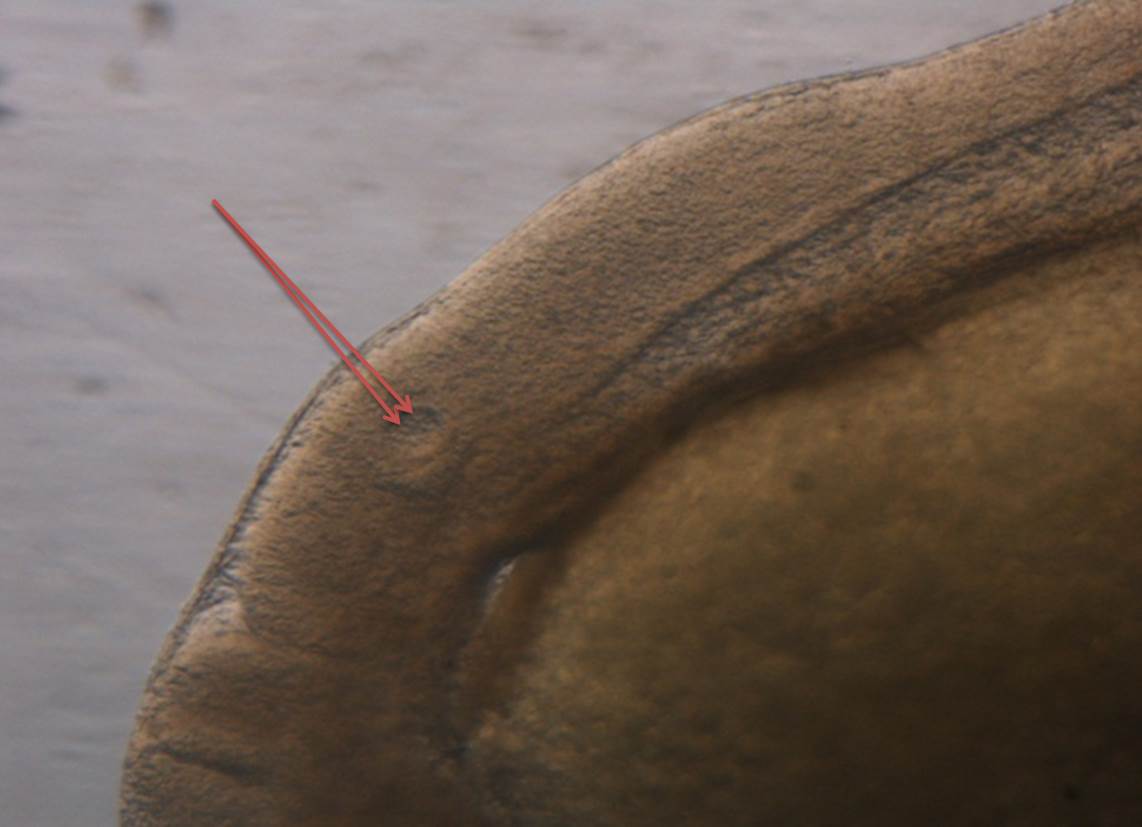

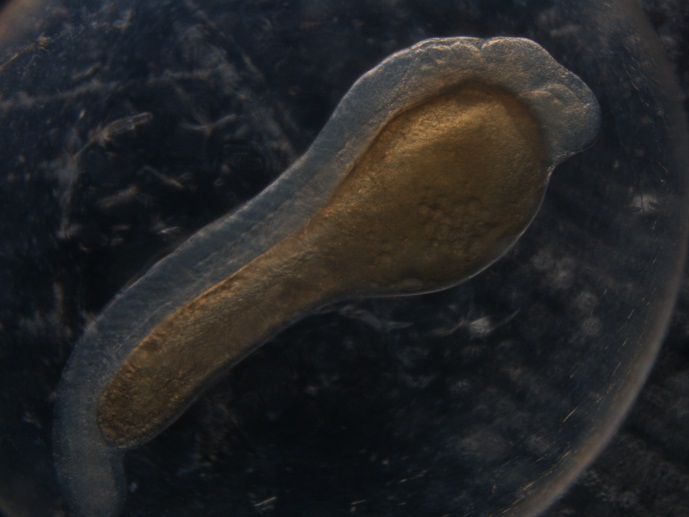

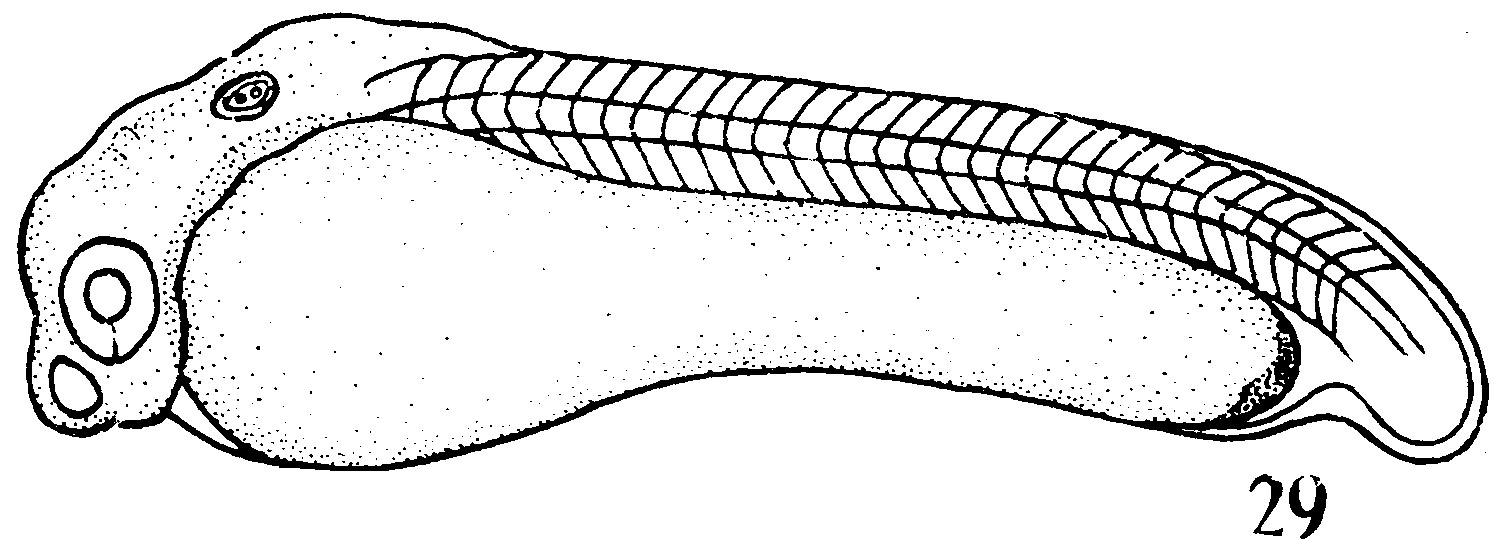


Figure 30) Otolith appearance stage (preserved, 112.5x; preserved, 4x). Arrows point to otoliths. Line drawing from Yi and others (1988).


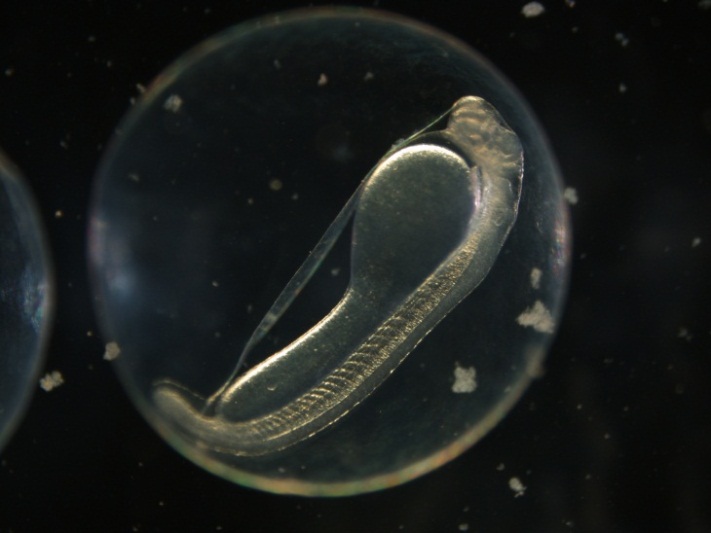

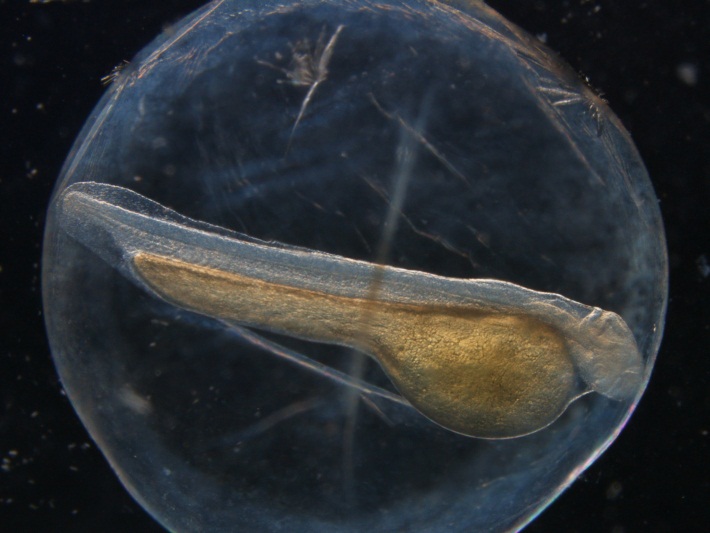


Figure 31) Heartbeat stage (Live, 30x; preserved, 30x). Line drawing from Yi and others (1988).


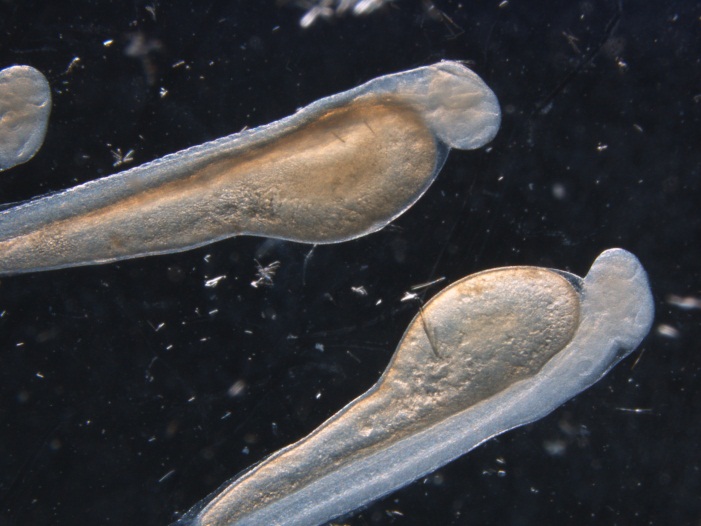

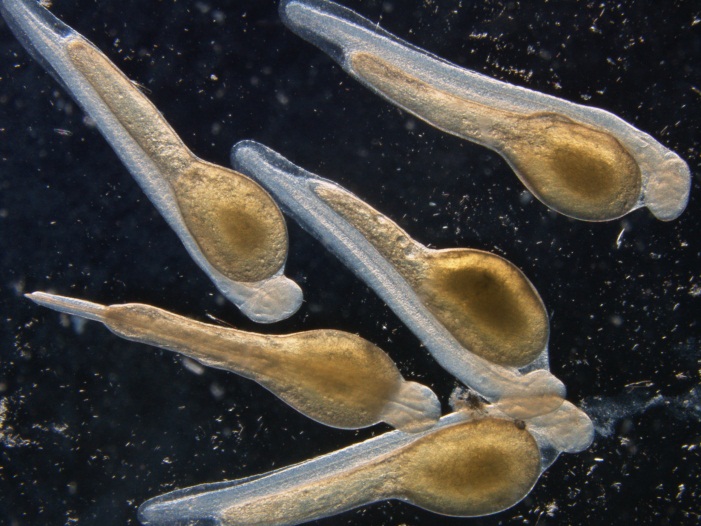

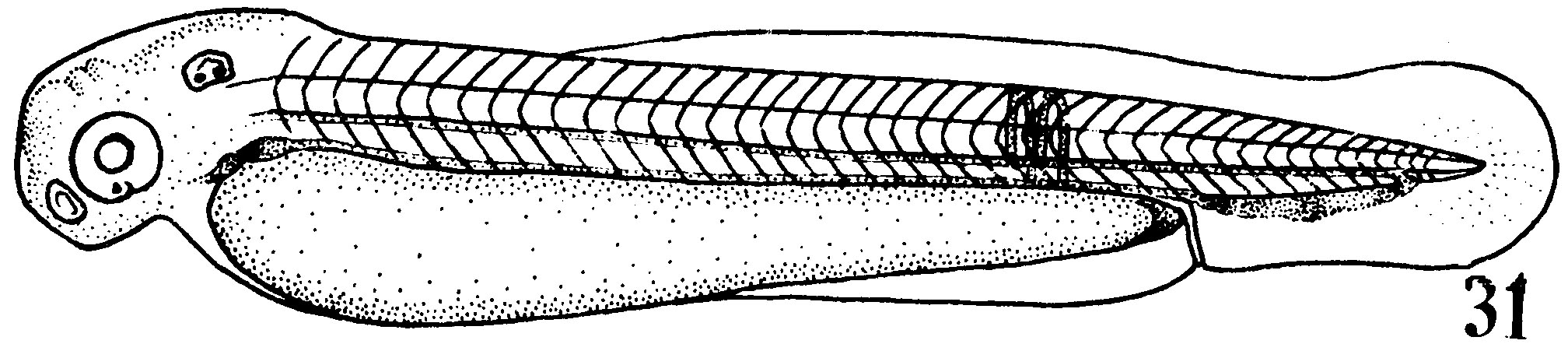


Figure 32) Hatching stage (preserved, 30x; preserved, 20x). Note lack of eyespots. Line drawing from Yi and others (1988).


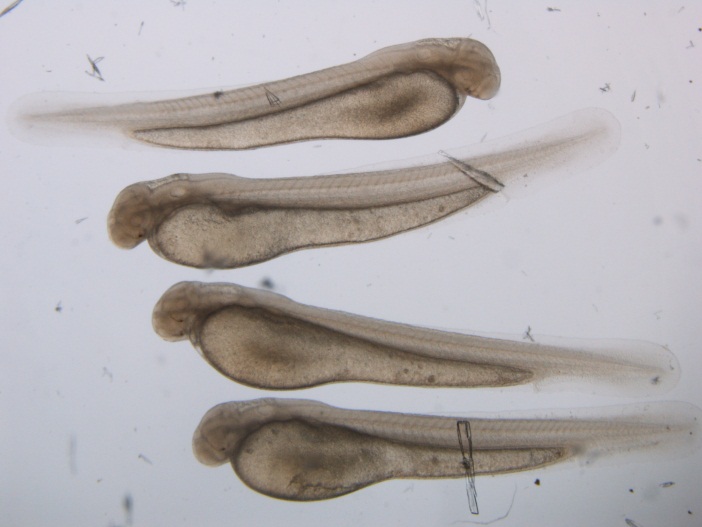

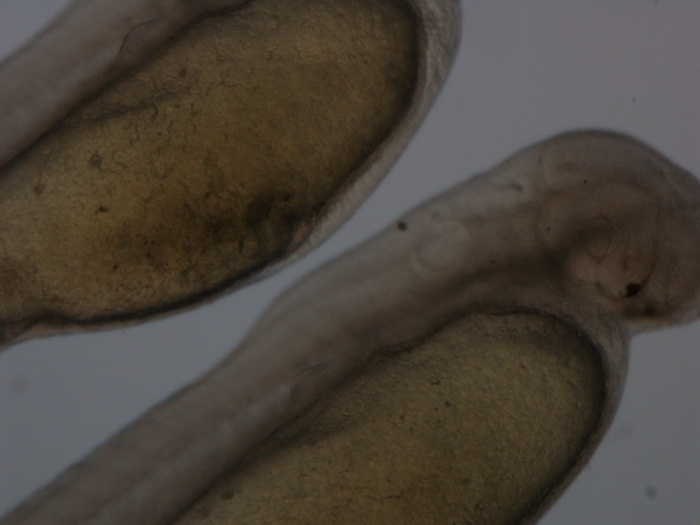

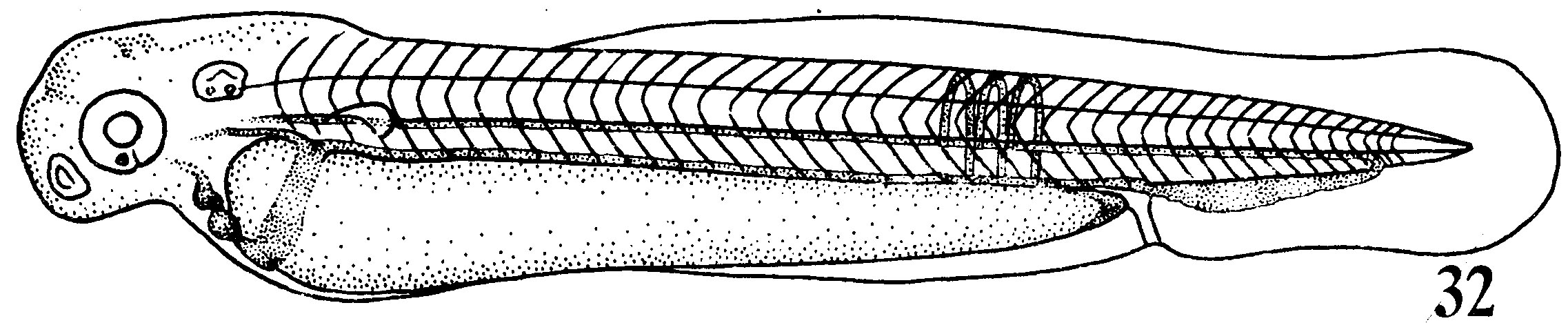


Figure 33) Rudimentary pectoral fin stage (Preserved, 20x; preserved, 70x). Line drawing from Yi and others (1988).


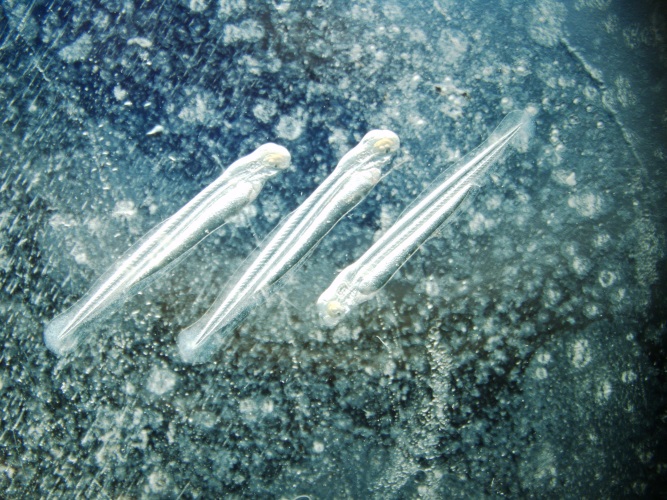

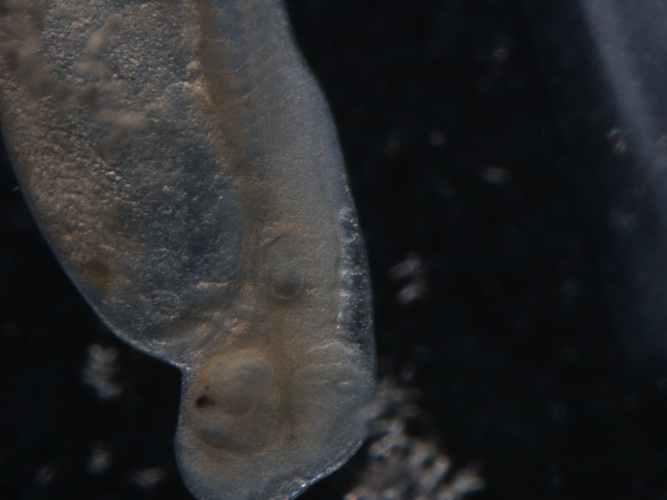

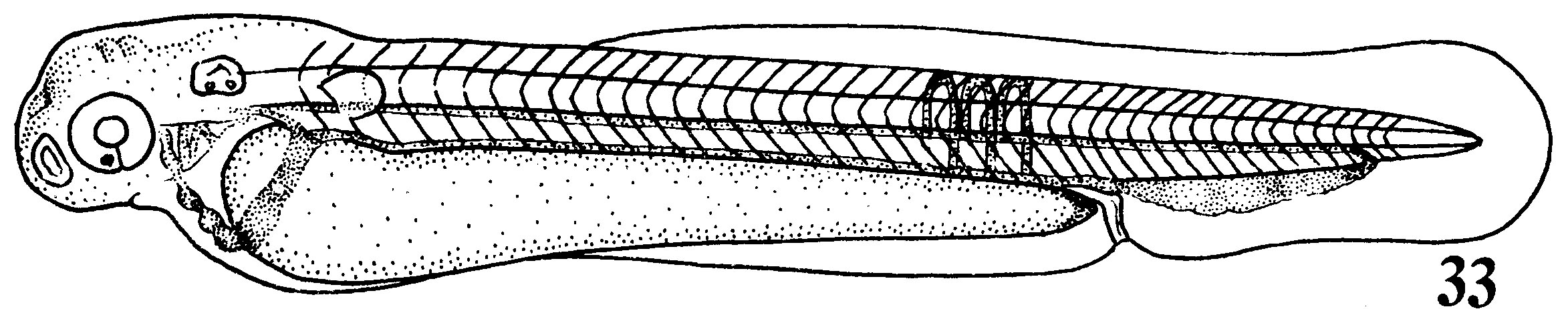


Figure 34) Gill arch stage (Live, 10x; preserved, 70x). Line drawing from Yi and others (1988).


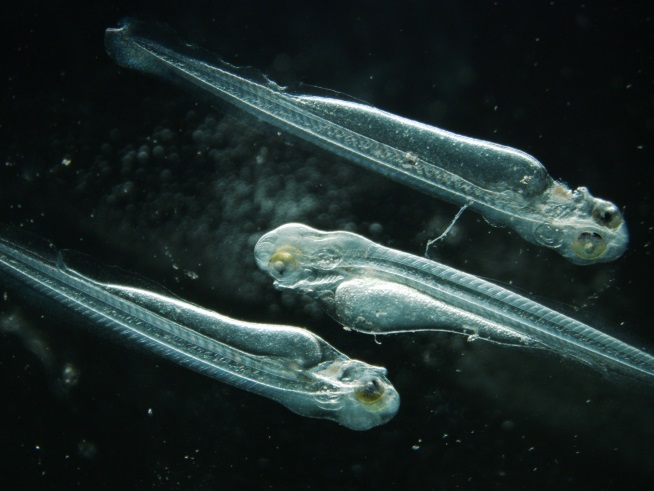

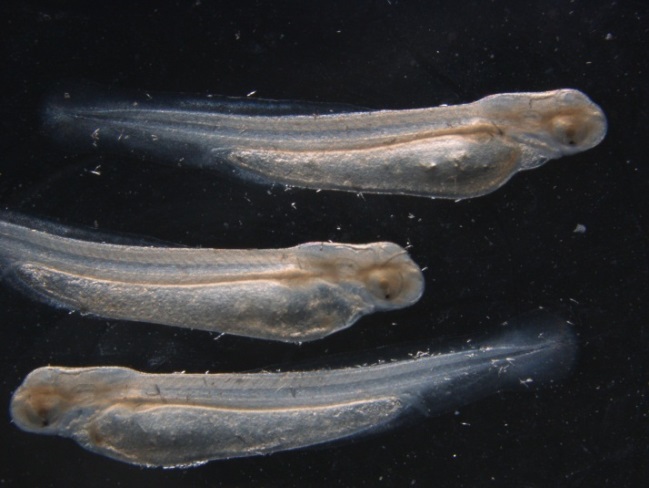

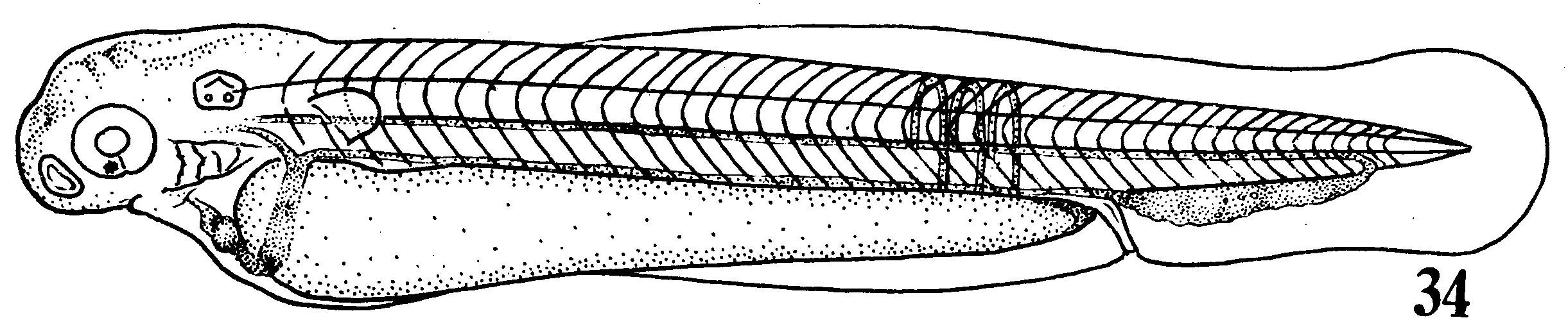


Figure 35) Xanthic eye stage (Live, 20x; preserved, 20x). Line drawing from Yi and others (1988).


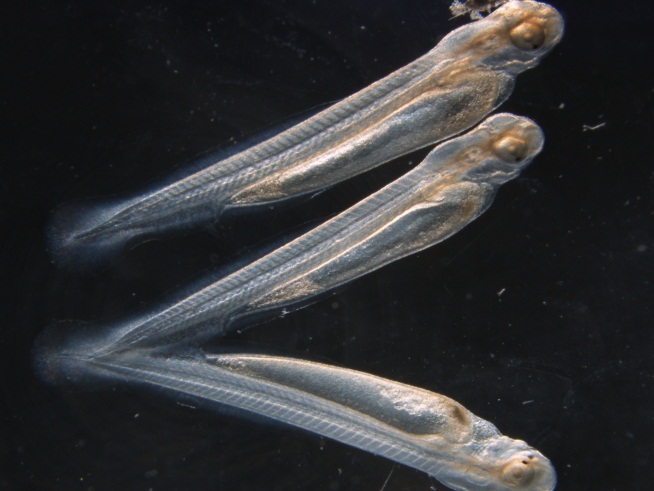

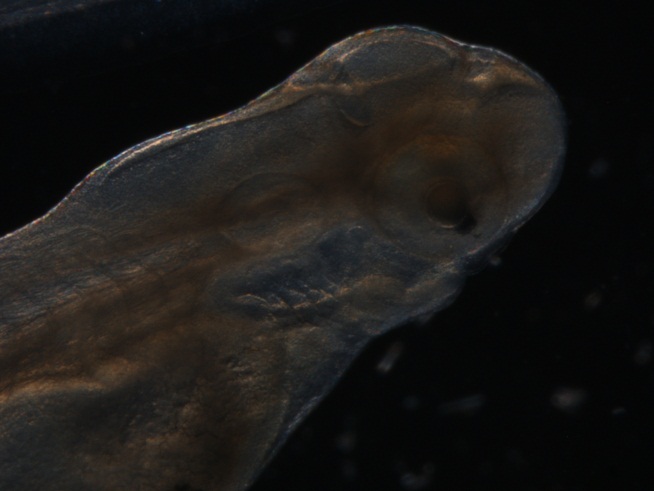


Figure 36) Gill filament stage (preserved, 20x; preserved, 80x). Line drawing from Yi and others (1988).

Figure 37) Melanoid eye stage (preserved, 40x, stage incomplete; preserved, 20x, stage complete). Line drawing from Yi and others (1988).

Figure 38) Gas bladder emergence stage (live, 40x; preserved, 20x). Line drawing from Yi and others (1988).

Figure 39) One chamber gas bladder stage (live, 10x; preserved, 20x). Line drawing from Yi and others (1988).

Figure 40) Yolk sac absorption stage (Live, 10x; preserved, 20x).

Figure 41) (Top to bottom) Comparison of grass carp, bighead carp, and silver carp larvae, at developmental stage 32-33 (preserved, 20x).
